# Too rigid to fold: Carotenoid-dependent decrease in thylakoid fluidity hampers the formation of chloroplast grana

**DOI:** 10.1093/plphys/kiaa009

**Published:** 2020-11-28

**Authors:** Michał Bykowski, Radosław Mazur, Joanna Wójtowicz, Szymon Suski, Maciej Garstka, Agnieszka Mostowska, Łucja Kowalewska

**Affiliations:** 1 Department of Plant Anatomy and Cytology, Institute of Plant Experimental Biology and Biotechnology, Faculty of Biology, University of Warsaw, Warsaw 02-096, Poland; 2 Department of Metabolic Regulation, Institute of Biochemistry, Faculty of Biology, University of Warsaw, Warsaw 02-096, Poland; 3 Laboratory of Electron Microscopy, Nencki Institute of Experimental Biology, Polish Academy of Sciences, Warsaw 02-093, Poland

## Abstract

In chloroplasts of land plants, the thylakoid network is organized into appressed regions called grana stacks and loosely arranged parallel stroma thylakoids. Many factors determining such intricate structural arrangements have been identified so far, including various thylakoid-embedded proteins, and polar lipids that build the thylakoid matrix. Although carotenoids are important components of proteins and the lipid phase of chloroplast membranes, their role in determining the thylakoid network structure remains elusive. We studied 2D and 3D thylakoid network organization in carotenoid-deficient mutants (*ccr1-1*, *lut5-1*, *szl1-1*, and *szl1-1npq1-2*) of Arabidopsis (*Arabidopsis thaliana*) to reveal the structural role of carotenoids in the formation and dynamics of the internal chloroplast membrane system. The most significant structural aberrations took place in chloroplasts of the *szl1-1* and *szl1-1npq1-2* plants. Increased lutein/carotene ratio in these mutants impaired the formation of grana, resulting in a significant decrease in the number of thylakoids used to build a particular stack. Further, combined biochemical and biophysical analyses revealed that hampered grana folding was related to decreased thylakoid membrane fluidity and significant changes in the amount, organization, and phosphorylation status of photosystem (PS) II (PSII) supercomplexes in the *szl1-1* and *szl1-1npq1-2* plants. Such changes resulted from a synergistic effect of lutein overaccumulation in the lipid matrix and a decreased level of carotenes bound with PS core complexes. Moreover, more rigid membrane in the lutein overaccumulating plants led to binding of Rubisco to the thylakoid surface, additionally providing steric hindrance for the dynamic changes in the level of membrane folding.

## Teaser

Increases in lutein/carotenoid ratios lead to decreased thylakoid fluidity and hamper grana folding due to carotenoid-dependent changes in both photosynthetic complexes and lipid matrix organization.

## Introduction

Carotenoids are lipophilic pigments; their biosynthesis undergoes dynamic changes during plant ontogenesis. They accumulate in chloroplasts of angiosperms (Lut, lutein; β-Car, β-carotene; Vio, violaxanthin; Neo, neoxanthin) and are necessary for various biological processes playing diverse roles, e.g. as signaling molecules and, due to their light-harvesting and photoprotective capacity, are essential for proper functioning of the photosynthetic apparatus (e.g. Havaux, 2014; Hashimoto et al., 2006; Liguori et al., 2017; Zhou et al., 2020).

In functional chloroplasts of fully developed plants, carotenoids are present in lipid and protein fractions of thylakoid membranes (Gruszecki and Strzałka, 2005; Dall’Osto et al., 2014). The majority of thylakoid carotenoids are bound to specific sites of photosynthetic complexes. The unbound carotenoid pool is freely dispersed in the lipid phase and constitutes around 15% of the total carotenoid content in Arabidopsis (*Arabidopsis thaliana*) chloroplasts (Dall’Osto et al., 2010).

Polar carotenoids localized in lipid phase have variable structures enabling their vertical or horizontal orientations within lipid bilayer dependent on particular molecule conformation. Such variable carotenoid orientation in the lipid phase, built mainly by monogalactosyldiacylglycerol (MGDG), digalactosyldiacylglycerol (DGDG), sulfoquinovosyldiacylglycerol (SQDG), and phosphatidylglycerol (PG), can cause changes in membrane rigidity and stability via van der Waals interactions between accessory pigments and polar lipid components (Gruszecki and Strzałka, 2005).

Carotenoids, which are present in chlorophyll–protein (CP) complexes as their integral components and linking molecules, play an important role in both photosystem (PS) reaction centers and are necessary for nonphotochemical quenching (NPQ), light-harvesting mechanisms, and protection against reactive oxygen species (reviewed in Hashimoto et al., 2006). Specific carotenoids predominantly occupy particular CP-complex components. Core complexes of both PSs are enriched with β-Car, while light-harvesting complexes (LHCs) predominantly bind Lut, Vio/zeaxanthin (Zea), and Neo (reviewed in Dall’Osto et al., 2014).

Thylakoid membranes built mainly by CP complexes and lipid matrix, both containing carotenoids, are folded into a 3D network of appressed (grana stacks) and nonappressed regions (stroma thylakoids). This type of organization links the ultrastructural level with a molecular one via the formation of an organized base for variable kinds of interactions between membrane components. The structural role of lipid and protein components in the formation of intricate spatial thylakoid system capable of adapting to changing environmental conditions has been a subject of many studies (e.g. Kirchhoff et al., 2004; Krumova et al., 2010; Armbruster et al., 2013; Garab et al., 2016; Yokoyama et al., 2016; Seiwert et al., 2018; Mazur et al., 2019; Wood et al., 2019; Albanese et al., 2020; Zsiros et al., 2020). Although carotenoids are essential and highly abundant components of thylakoids, their role in the formation and plasticity of the structure of chloroplast membrane network has not been elucidated yet. Localization of carotenoids in two distinct thylakoid regions makes it difficult to reveal the direct structural role of carotenoids due to the synergistic effect of their presence in the lipid and protein fraction of thylakoids.

We used Arabidopsis mutants with decreased/increased levels of particular xanthophylls and carotenes: *ccr1-1*, *lut5-1*, *szl1-1*, *szl1-1npq1-2* to decipher how the formation of the thylakoid network structure is affected by altered levels of different carotenoids. The relative decrease of the Lut content in the *ccr1-1* mutant is related to a lowered expression of the *CAROTENOID ISOMERASE* (*CRTISO*) catalyzing lycopene isomerization (Cazzonelli et al., 2009). Altered carotenoid levels in the *lut5-1* mutant are caused by the mutation of *LUTEIN DEFICIENT 5* (*LUT5*) gene coding the β-ring hydroxylase (Kim and DellaPenna, 2006). Therefore, all hydroxycarotenoid levels are reduced, β-Car mostly. However, the total Car pool is not decreased significantly due to an overaccumulation of α-Car molecules (Dall’Osto et al., 2007). In the *szl1-1* and *szl1-1npq1-2* mutants, the increase in the Lut level is related to the mutation of *SUPRESOR OF ZEAXANTHIN-LESS 1* (*SZL1*) gene coding lycopene β-cyclase (LYC-B), which impairs more severely β-β-xantophyll than the β-ε-xantophyll branch of the carotenoid biosynthesis pathway (Li et al., 2009; Cazzaniga et al., 2012). Additionally, the *szl1-1npq1-2* mutant has the mutation in violaxanthin deepoxidase coded by *NON-PHOTOCHEMICAL QUENCHING 1* (*NPQ1*) gene, which results in the complete lack of Zea in all light conditions (Li et al., 2009). Due to high redundancy in the enzymes of carotenoid biosynthesis pathways, most of the carotenoid-deficient mutants reveal only partial depletion in the levels of particular carotenoids showing a disturbed balance between different carotenoid groups.

The main goal of this study was to clarify the structural role of different carotenoids in the formation and plasticity of the ultrastructure and spatial arrangement of the thylakoid network. We established that the proper Lut/Car ratio is particularly important to maintain efficient thylakoid membrane folding. Carotenoid-dependent reduction in grana size was related to decreased thylakoid membrane fluidity and altered interactions of PS complexes. This resulted from the synergistic effect of Lut overaccumulation and decrease of the Car level, both considerably influencing properties of the thylakoid lipid phase as well as the abundance of the photosynthetic proteins, their organization, and phosphorylation status.

## Results

To determine the structural role of particular carotenoids in the formation of the thylakoid network arrangement, plants of selected carotenoid-deficient mutants were grown in low light (LL) conditions (70 *µ*mol photons m^−2 ^s^−1^), which were nondestructive for all tested genotypes. Moreover, 120 *µ*mol photons m^−2^ s^−1^ light intensity, excessive light (EL) condition for LL-grown plants, were applied during the final week of growth.

### Plant phenotype and carotenoid composition

**Figure 1:**
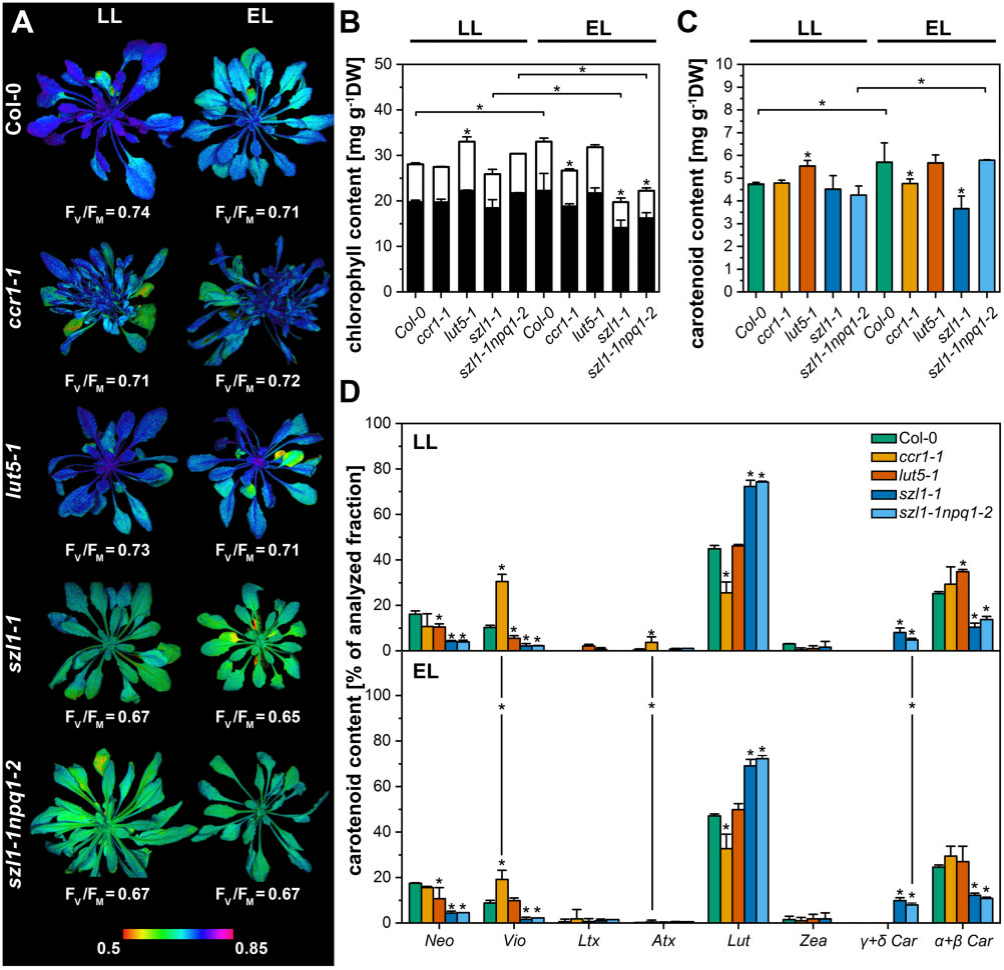
Characterization of 10-week-old Columbia-0 (Col-0) and carotenoid-deficient Arabidopsis (*A. thaliana*) plants grown in LL (70-*µ*mol photons m^−2^ s^−1^) and adapted to EL conditions (120-*µ*mol photons m^−2^ s^−1^). A, Chl *a* fluorescence imaging analyses. Images present rosette morphology and the distribution of the maximal quantum yield of PSII (F_V_/F_M_) values. B, Chl content per dry weight tissue (DW). Chl *a*, black parts of bars; Chl *b*, white parts of bars. C, Carotenoid content per DW. D, Particular carotenoid (Neo, Vio, Ltx, Atx, Lut, Zea, Car) percentage contribution in the total carotenoid pool. Data are mean values ± sd from at least three independent experiments. Results marked with an asterisk on the top of the bar differ significantly at *p *=* *0.05 (one-way ANOVA with post hoc Tukey test) from Col-0 in respective light condtions. Asterisk on the top of the line indicates significant differences in the results of a particular genotype between LL and EL conditions. Images of plants shown in (A) were digitally extracted for comparison.

The phenotypes of analyzed mutants were similar, except for the *ccr1-1* mutant with increased shoot branching, which was consistent with literature data (Cazzonelli et al., 2009; Figure 1). Chlorophyll (Chl) *a* fluorescence imaging data revealed a decreased maximal efficiency of PSII (F_V_/F_M_) levels in *szl1-1* and *szl1-1npq1-2* compared to other examined plants in both light conditions (Figure 1A). However, the content of the Chl per dry weight in both Lut overaccumulating mutants (*szl1-1* and *szl1-1npq1-2*) was lower compared with Col-0 plants in EL conditions only. In LL conditions, Chl and the total carotenoid contents per dry weight were similar in all examined genotypes except of higher values registered in *lut5-1* plants (Figure 1, B and C).

The *ccr1-1* mutant exhibited a substantial decrease in the Lut and increase in Vio contribution in the total carotenoid fraction (Figure 1D). The *lut5-1* mutant showed a decrease in Neo and Vio content with a simultaneous increase in β-Car and α-Car contribution compared with wild-type (wt; Figure 1D). UPLC analyses revealed highly increased Car-α/Car-β ratio in the *lut5-1* mutant (Supplemental Table S1), which is consistent with the literature data (Kim and DellaPenna, 2006). Some increase in the α-Car/β-Car ratio was also detected in both Lut overaccumulating mutants together with more pronounced changes in different carotenoid classes compared with Col-0 plants (Figure 1D, Supplemental Table S1). The presence of two additional peaks in chromatograms with absorption maxima at 430–455–486 nm and 433–458–486 nm were registered in both Lut overaccumulating mutants and recognized as δ-Car and γ-Car (Supplemental Figure S1). However, in the *szl1-1* and *szl1-1npq1-2* plants, the Lut/Car total (α + β+δ + γ) ratio remained significantly increased compared to other examined genotypes (Supplemental Table S1). Moreover, the total lack of Zea was registered in the *szl1-1npq1-2* plants only. EL conditions resulted in a decrease in Vio level in the *ccr1-1* plants (Figure 1D) and induced a decrease in the Car-α/Car-β ratio in the *lut5-1* mutant (Supplemental Table S1).

### Thylakoid network ultrastructure is modified in carotenoid-deficient plants

**Figure 2:**
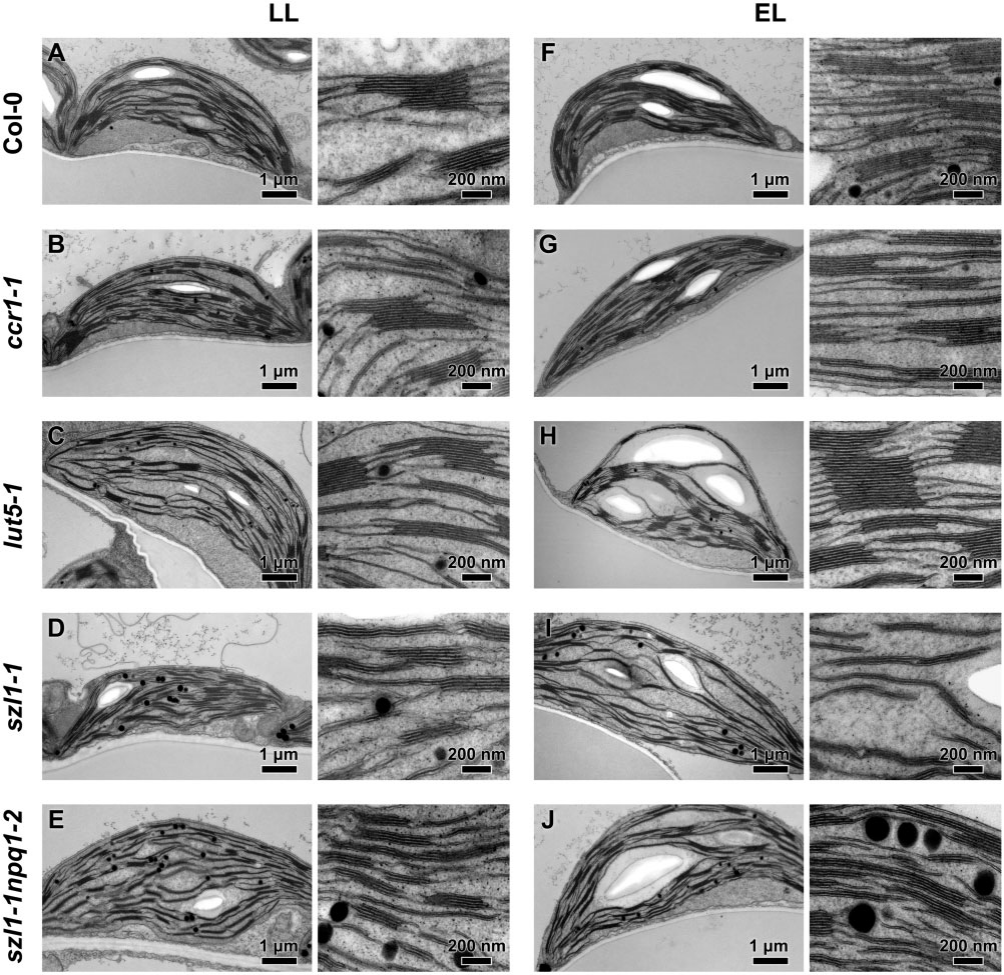
Electron micrographs of mesophyll chloroplasts of Columbia-0 (Col-0) and carotenoid-deficient Arabidopsis (*A. thaliana*) plants grown in LL and adapted to EL conditions. A–J, Exemplar chloroplast images (left side of each panel) and thylakoid network enlargements (right side of each panel) of all analyzed genotypes.

**Figure 3:**
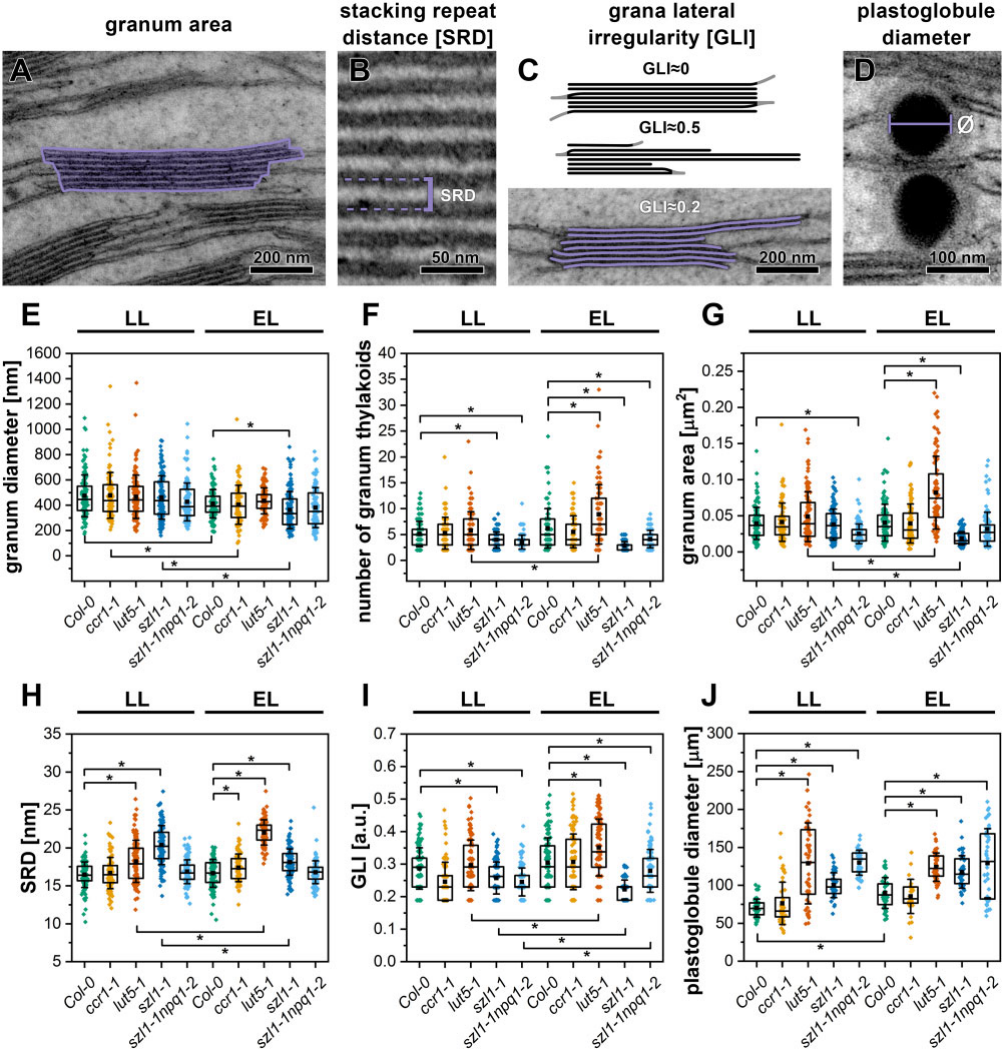
Quantitative changes in key structural parameters of the thylakoid network measured from electron micrographs of mesophyll chloroplasts of Columbia-0 (Col-0) and carotenoid-deficient Arabidopsis (*A. thaliana*) plants grown in LL and adapted to EL conditions. A–D, Graphical representation of selected structural features and the thylakoid membrane parameters measured and calculated from electron micrographs. E, Granum diameter. F, Number of thylakoids building a granum stack. G, Cross-sectional granum area. H, SRD. I, GLI. J, Cross-sectional plastoglobule diameter; the bottom and top of each box represent 25 and 75 percentile, respectively. The whiskers denotes standard deviation (SD), every point visible on the boxes represent individual measurement; pairs of results marked with asterisk differ significantly at *p *=* *0.05 (one-way ANOVA with post hoc Tukey test; *n* = 90–144); only differences between mutants and Col-0 in respective light conditions and for particular genotypes in both light conditions are marked.

Chloroplast of LL-grown genotypes exhibited typical ultrastructure, characteristic for mature chloroplast of higher plants (Figures 2–3). However, we observed a significant decrease in the number of grana stacks per *µ*m^2^ in the *szl1-1* plants (Supplemental Figure S2A). In LL conditions, we did not register any substantial differences in diameters of grana between examined plants (Figure 3E), while the area of grana cross-sections was smaller in the *szl1-1npq1-2* mutant only (Figure 3, A and G). The *szl1-1-npq1-2* mutant had also the smallest grana height as well as number of membrane layers per granum stack, as opposed to the highest stacks observed in the *lut5-1* mutant (Figure 3F, Supplemental Figure 2B). This was mainly due to the higher values of stacking repeat distance (SRD; Figure 3B) in the *lut5-1* mutant rather than an increased number of thylakoid layers in grana stacks compared to wt plants (Figure 3, F and H). The value of the SRD parameter increased significantly in the *szl1-1* mutant compared to wt plants (Figure 3H) and was the reason for similar Col-0 values of grana height (Supplemental Figure S2B) registered together with a decrease in the number of thylakoids per granum stack (Figure 3F). One of the essential grana structural parameters is grana lateral irregularity (GLI) describing how particular grana membranes fit the hypothetical cylinder—the lower the value, the more regular the grana stack (Figure 3C). The lowest GLI values were reached in the *szl1-1npq1-2* and *sz1-1* plants (Figure 3I). Moreover, we calculated how cross-sectional grana sizes and their regularity influence the ratio of the length of granum end-membranes to the total length of stacked thylakoids building the particular granum (Supplemental Figure S2D). End-membranes to the total granum thylakoids lengths were increased in the *szl1-1npq1-2* plants only, which correlates with the smallest grana stacks registered in this mutant in LL conditions (Supplemental Figure S2E). Moreover, from chloroplast transmission electron microscopy (TEM) cross-sections we calculated the number and diameter of plastoglobules (Figure 3, D and J, Supplemental Figure S2C). The highest number per *µ*m^2^ and the largest diameter of plastoglobules were registered in the *szl1-1npq1-2* plants; large plastoglobule diameters were also observed in the *szl1-1* and *lut5-1* mutants compared with Col-0 (Figure 3J).

Plants exposed to EL showed a decrease of grana diameters compared to the LL condition with most pronounced changes in wt and the *szl1-1* plants (Figure 3E). However, the overall cross-sectional granum area in EL conditions was decreased in the *sz1-1* plants only, where EL also caused a decrease of grana height, mainly due to the significant change in the SRD value (Figure 3, F–H, Supplemental Figure S2B). The decrease in the size of *szl1-1* grana stacks in EL conditions correlates with a significant increase in the number of grana per *µ*m^2^ and an increase in the end/total granum thylakoids ratio suggesting that grana stacks of this mutant undergo a horizontal division in EL conditions (Supplemental Figure S2A, E). A significant increase in the grana height and area registered in the *lut5-1* mutant in EL conditions (Figure 3G, Supplemental Figure S2B) was due to the multiplication of granum thylakoid layers and an increase in the SRD value (Figure 3, F and H). The increase of the GLI value in the *lut5-1* plants was associated with a notably larger grana registered in this mutant in EL conditions (Figure 3I). EL also caused an increase in the number and size of plastoglobules in the *szl1-1npq1-2* and Col-0 plants, respectively (Figure 3J, Supplemental Figure S2C).

### Carotenoid composition affects lens-like shape of the thylakoid network

**Figure 4:**
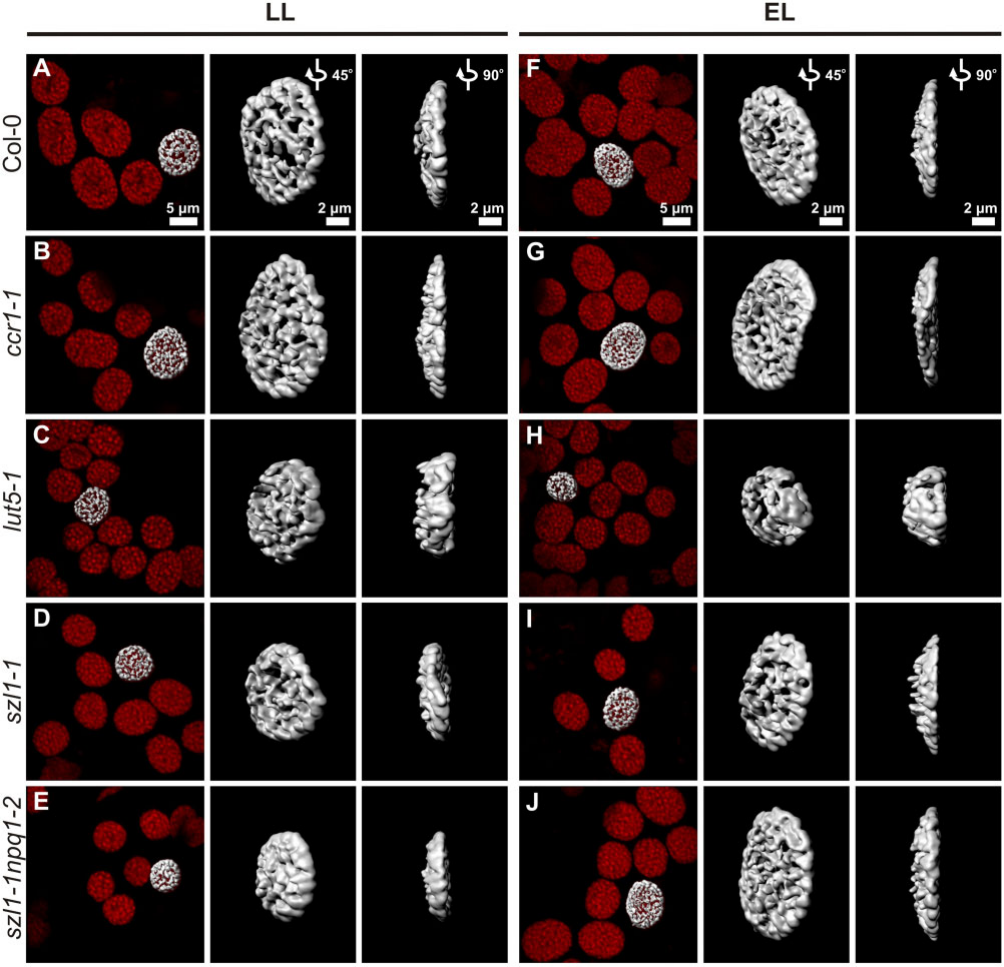
Volume of Chl fluorescence of mesophyll cell chloroplasts of Columbia-0 (Col-0) and carotenoid-deficient Arabidopsis (*A. thaliana*) plants grown in LL and adapted to EL conditions, as well as 3D reconstruction of the fluorescence surface reflecting thylakoid network shape. A–J, Exemplar images of Chl fluorescence of chloroplasts visible inside living mesophyll cell with isosurface generated for selected chloroplasts (left side of each panel), 3D generated isosurface of Chl autofluorescence of single chloroplast presented from two different angles (middle and right side). Scale bars shown in images in (A) and (F) apply to all images in the same column.

**Figure 5:**
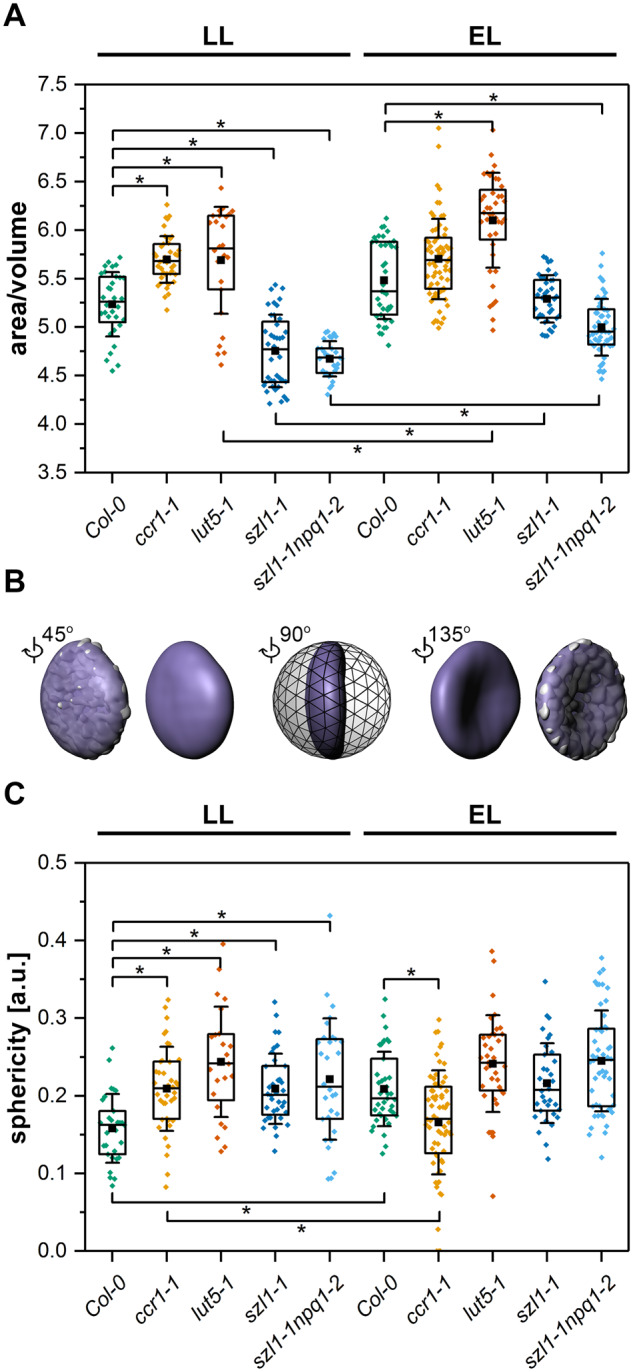
Spatial parameters of thylakoid network shape and level of folding in chloroplasts of Columbia-0 (Col-0) and carotenoid-deficient Arabidopsis (*A. thaliana*) plants grown in LL and adapted to EL conditions. A, Ratio of area of 3D generated Chl fluorescence isosurface to its inner volume indicating level of the overall thylakoid network folding. B, Graphical representation of the simplified surface of the Chl fluorescence, revealing the lens-like shape of the thylakoid network (purple) superimposed on the actual Chl fluorescence area model (light gray). Models are visible from different angles and presented in the context of a sphere with the radius identical to the semi-major axis of a simplified thylakoid model. Such comparison of a sphere and simplified thylakoid model volumes is described as a sphericity parameter; the higher the value the more the thylakoid network shape is similar to the sphere with the same diameter as the major axis of a given object. C, Sphericity parameter calculated as described above; description of the chart boxes as on Figure 3. Pairs of results marked with asterisk differ significantly at *p *=* *0.05 (one-way ANOVA with post hoc Tukey test; *n* = 24–66).

To reveal the spatial (3D) shape of the chloroplast thylakoid network, we performed confocal laser scanning microscopy (CLSM) analysis using *in vivo* approach (Figure 4). Red spots are attributed to the Chl bound to PSII, which corresponds to grana positions inside chloroplasts. Based on 3D Chl fluorescence volumes, we generated isosurface models and calculated different structural parameters from them (Figures 4–5). The ratio of the area to the volume of the generated model (A/V) reflects the degree of folding of the whole thylakoid network and therefore combines the size and distribution of grana stacks inside the chloroplast. In LL conditions, the *ccr1-1* and *lut-5-1* mutants exhibited increased A/V values while overaccumulating Lut plants showed the opposite effect compared with wt plants (Figure 5A). EL conditions caused an increase of A/V values in the *lut5-1*, *szl1-1*, and *szl1-1npq1-2* plants compared to the LL growth (Figure 5A). Thylakoid network shape visualized in CLSM in vivo can indirectly characterize the physical properties of membrane arrangements and might be described as the “sphericity” parameter that allows to distinguish between flattened, typical lens-like (Figure 5B) and swollen shape of the thylakoid network. All examined mutants grown in LL conditions exhibited higher sphericity values, *lut-5-1* in particular, compared with Col-0 plants (Figure 5C). EL conditions affected mostly the sphericity of wt plants in which the registered values reached levels observed in mutants, except for significantly flattened thylakoid network of EL-grown *ccr1-1* plants (Figure 5C).

### Macro-domain arrangement of pigment–protein complexes is severely affected in lut overaccumulating mutants

**Figure 6:**
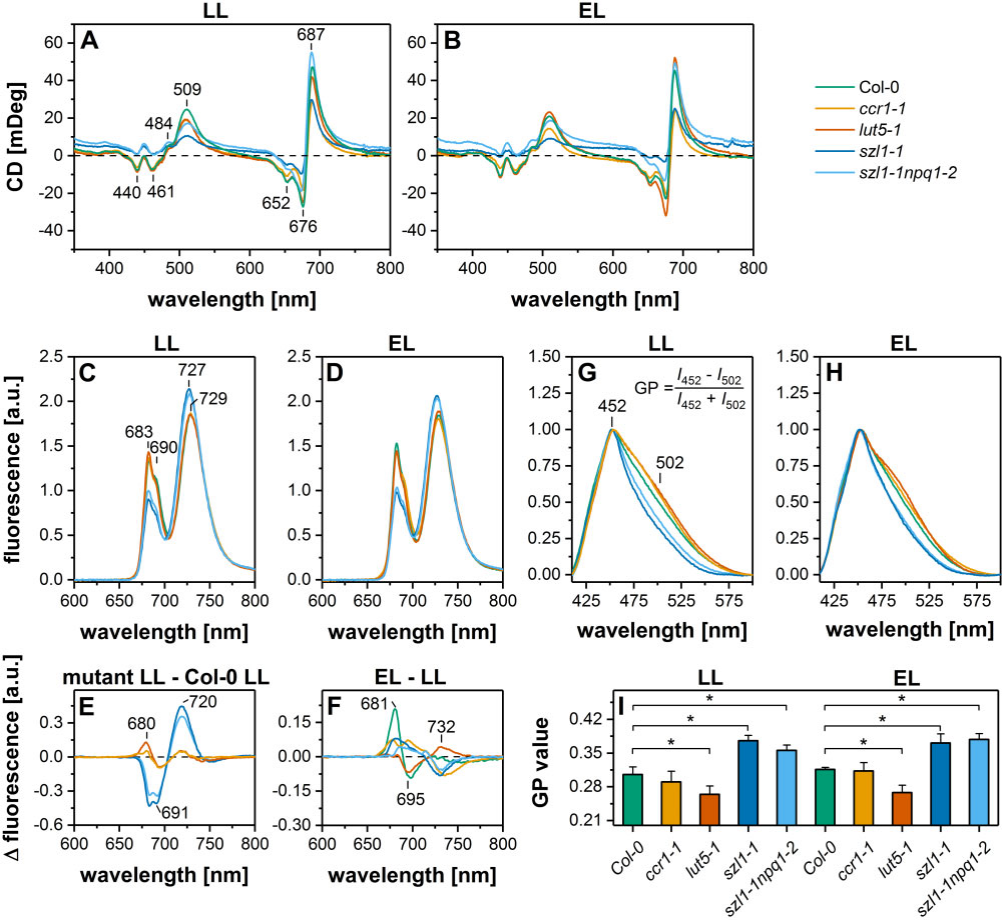
Spectroscopic analysis of thylakoids isolated from Columbia-0 (Col-0) and carotenoid-deficient Arabidopsis (*A. thaliana*) plants grown in LL and adapted to EL conditions. A, B, CD spectra normalized to the maximum of the Chl Q_y_ absorption band. C, D, Low temperature (77 K) chlorophyll fluorescence emission spectra normalized to equal area under the curve. E, F, Chlorophyll fluorescence emission difference spectra mutant-minus-Col-0 in LL conditions (E) and EL–LL for all genotypes (F). G, H, Laurdan fluorescence emission spectra normalized to the maximal value. I, Laurdan GP values for LL and EL samples. All presented spectra are representative of three independent experiments; pairs of results marked with asterisk differ significantly at *p* = 0.05 (one-way ANOVA with post hoc Tukey test; *n* ≥ 4).

Different types of macro-domains formed by pigment–protein complexes are higher order structures playing a role in the formation of the thylakoid membrane ultrastructure. The CD Ψ-type band with a maximum at around (+)509 nm corresponds to carotenoids embedded in long-range ordered structures (Tóth et al., 2016). In LL conditions, the highest signal of 509 nm band was registered in Col-0 thylakoids; lower values were detected in all examined mutants, in the *szl1-1* mutant in particular (Figure 6A). EL induced a decrease in the amount of long-range ordered structures containing carotenoids in Col-0 and *ccr1-1* plants. The opposite effect was detected in the thylakoids of *lut5-1* mutant (Figure 6B). Substantial differences were also registered in the amplitudes of the (-)652 nm band, as well as bands between 400 nm and 461 nm, originating from short-range Chl excitonic interactions (Tóth et al., 2016), which were substantially lower in the Lut overaccumulating mutants compared to other examined genotypes (Figure 6, A and B).

### Increased Lut/Car ratio causes substantial changes in the supramolecular organization of CP complexes and thylakoid membrane fluidity

Low-temperature Chl fluorescence spectra revealed a significant decrease in the contribution of PSII complexes (683 and 690 nm) and an increase of the PSI complexes (727–729 nm) in the emission spectra of thylakoids of the Lut overaccumulating mutants compared to other examined plants (Figure 6C). Moreover, only in the *szl1-1* and *szl1-1npq1-2* plants the band corresponding to PSI complexes was shifted to blue light (727 nm). The difference spectra revealed a broad positive peak at 720 nm, suggesting that the observed shift was probably related to the formation of PSI–LHCI–LHCII complex (Figure 6E; Chuartzman et al., 2008). EL induced most pronounced changes in the spectra of Col-0 plants—an increase in the signal originating from PSII supercomplexes and a decrease in the level of PSII core complexes (Figure 6, D and F).

Chl fluorescence excitation spectra were recorded at 77 K for emissions at 685 and 735 nm, which are characteristic for Chls bound to PSII and PSI, respectively (Supplemental Figure S3). PSII excitation spectra showed significantly lower values in the range characteristic for Chl *b* and carotenoids attached to LHCII (around 466 and 488 nm negative peaks in difference spectra) in both Lut overaccumulating plants (Supplemental Figure S3A, C) pointing to a limited transfer of excitation from antennae to PSII core complexes in these mutants. EL caused the most noticeable changes in the Col-0 spectra visible as an increase in the signal originating from Chl *a* bound with PSII complexes (positive peaks at 410 and 433 nm in Col-0 EL–minus–Col-0 LL spectrum; Supplemental Figure S3D) as well as an increase in the signal characteristic for PSI-bound Chls (Supplemental Figure S3H) compared with LL conditions.

Spectroscopic analysis was also performed to establish thylakoid membrane fluidity (Figure 6, G and H). We registered signals from fluid- and gel-phase embedded laurdan fluorescent dye (6-dodecanoyl-N,N-dimethyl-2-naphthylamine) to calculate generalized polarization (GP) values; the higher the GP value the lower is membrane fluidity (Parasassi et al., 1990, Szilágyi et al., 2008). We established that in both light conditions thylakoid membranes of the *lut5-1* mutant were more fluid compared with respective Col-0 plants. In contrast, *szl1-1* and *szl1-1npq1-*2 thylakoid membranes were significantly less fluid compared with wt (Figure 6I).

**Figure 7:**
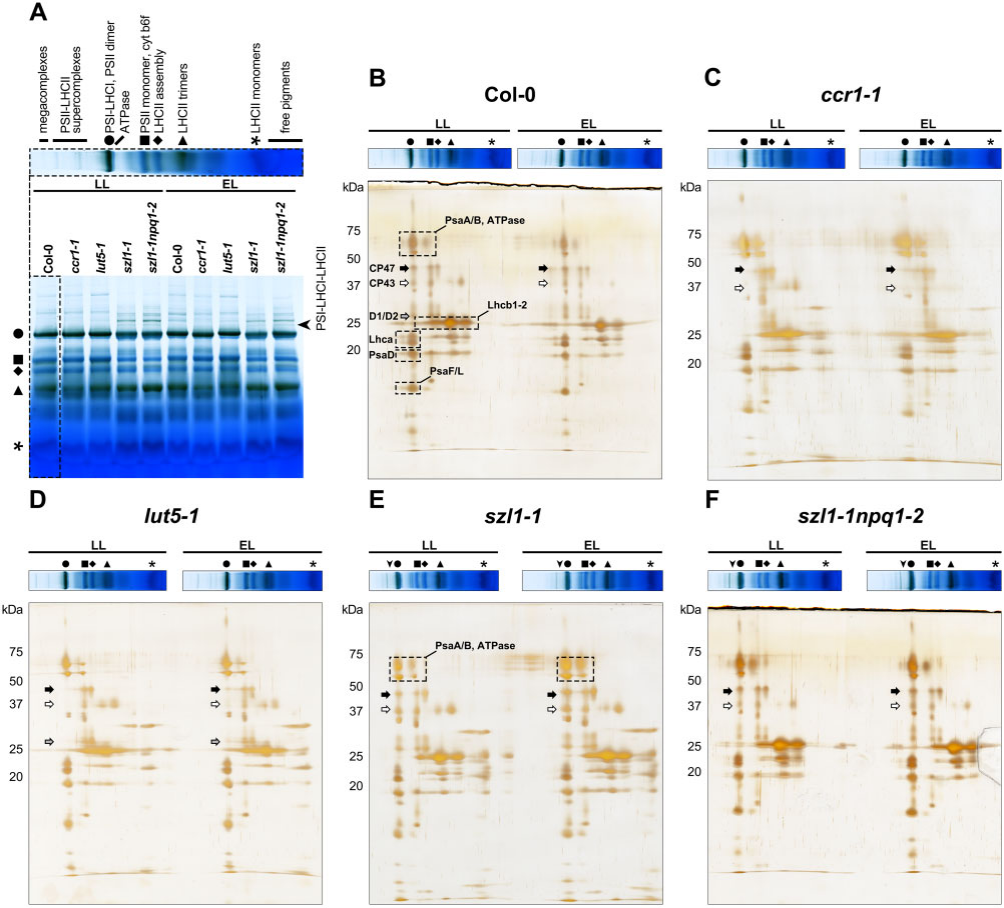
Analysis of arrangement and composition of CP complexes of thylakoids isolated from Columbia-0 (Col-0) and carotenoid-deficient Arabidopsis (*A. thaliana*) plants grown in LL and adapted to EL conditions. A, Blue native (BN) polyacrylamide gel electrophoresis (BN-PAGE) of CP complexes. B–F, Silver stained 2D-denaturing PAGE of BN-PAGE. Geometrical symbols indicate position of BN-PAGE bands described in panel A. All the presented data are representative of three independent experiments.

Organization of CP complexes was further assessed using the BN-electrophoresis method (Figure 7). We established that the arrangement of PSII supercomplexes was severely altered in the *szl1-1* and *szl1-1npq1-2* mutants by the presence of an increased amount of low-mass supercomplexes compared to all other examined genotypes (Supplemental Figure S4A). In both Lut overaccumulating plants, the presence of an additional band corresponding to PSI–LHCI–LHCII was registered (Figure 7A). Moreover, the *szl1-1* and *szl1-1npq1-2* plants exhibited a decreased amount of LHCII trimers, cytochrome *b*_6_*f*, PSI–LHCI complex, PSII dimer and monomer, and also slightly increased amount of monomers compared to wt plants (Supplemental Figure S4A). EL conditions affected mostly the organization of the Col-0 PSII–LHCII supercomplexes together with the increase of their amount, and also resulted in a decrease of the level of PSII–LHCII supercomplexes in the Lut overaccumulating plants compared to LL conditions (Figure 7A, Supplemental Figure S4B). Moreover, an increase in all lower-weight BN band intensities was detected in EL conditions in the *szl1-1* and *szl1-1npq1-2* mutants only (Figure 7A, Supplemental Figure 4B).

Finally, due to the fact that the level of phosphorylation of thylakoid proteins is frequently associated with the specific organization of CP complexes, we analyzed the abundance of phosphoproteins in the isolated thylakoid fractions (Supplemental Figure S5). Almost complete retardation of CP43, D1, D2, and PsbH protein phosphorylation with an increased level of phosphorylated Lhcb1 and Lhcb2 was detected in the Lut overaccumulating plants compared with Col-0 in both light conditions (Supplemental Figure S5).

### Thylakoid protein composition is significantly altered in plants with a disturbed carotenoid levels

**Figure 8:**
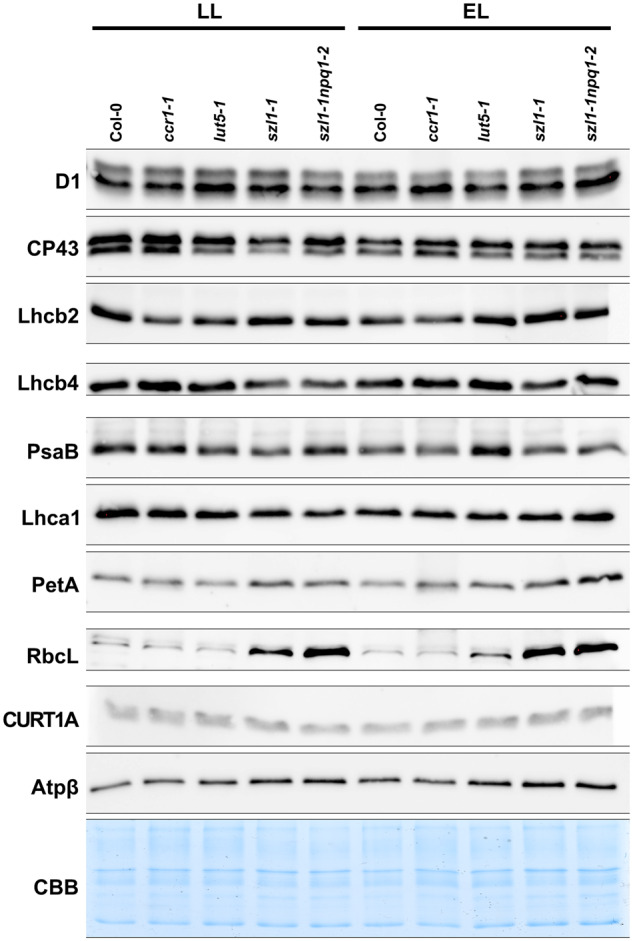
Immunodetection analysis of selected thylakoid proteins in thylakoid samples isolated from Columbia-0 (Col-0) and carotenoid-deficient Arabidopsis (*A. thaliana*) plants grown in LL and adapted to EL conditions. Western blot immunodetection of PSII core (D1 (AS10 704), CP43 (AS11 1787)) and antennae proteins (Lhcb2 (AS01 003), Lhcb4 (AS04 045)); photosystem I (PSI) core (PsaB (AS10 695)) and antennae (Lhca1 (AS01 005)) proteins; cytochrome b_6_f subunit (PetA (AS08 306)); Rubisco large subunit (RbcL (AS03 037)), CURT1A (AS08 316) and Atpβ (AS05 085) subunit of ATP-ase. CBB: Coomassie Brilliant Blue staining. All presented data are representative of three independent experiments.

The 2D-SDS-PAGE protein electrophoresis revealed the composition details of the particular BN-PAGE-separated photosynthetic complexes (Figure 7, B–F). In the *ccr1-1* and *lut5-1* mutants, a decreased amount of the PSII dimer was detected compared with other examined plants (Figure 7, B–F). Analyses of the 1D SDS electrophoretic pattern did not indicate any major changes between analyzed genotypes apart from an increase in LHCII amount in *lut5-1* compared to other examined plants (Supplemental Figure S4C). Further immunodetection revealed that the overall level of CP43, Lhcb4, and PsaB proteins was decreased in the *szl1-1* mutant compared with Col-0 plants (Figure 8). A significant decrease in the level of Lhcb4 protein was also noted in the *szl1-1npq1-2* mutant (Figure 8).

In Col-0 and *ccr1-1*, the EL conditions induced a decrease in the CP43 and PsaB level. (Figures 7, B and C and 8). In the *lut5-1* mutant, we observed a decrease in D1 (visible also in 2D-PAGE analysis—Figure 7D) with a simultaneous increase in the PsaB protein levels (Figure 8). In the *szl1-1npq1-2* mutant, however, the opposite effect was detected together with a decrease in the PsaB level and an increase in the level of PetA (Figure 8). The level of CURVATURE THYLAKOID 1A (CURT1A) was similar in all examined plants in both conditions, while the Atpβ was slightly increased in the Lut overaccumulating mutants. Immunodetection revealed a significant increase in the RbcL level in the thylakoid membrane fraction of the *szl1-1* and *szl1-1npq1-2* plants compared with other examined genotypes (Figure 8, Supplemental Figure S4E). To reveal whether these changes might be related not only to the increased Lut level but also to the difference in the polar lipid composition, we applied thin layer chromatography (TLC) method to track changes in the main thylakoid lipid matrix components (MGDG, DGDG, SQDG, and PG) in studied genotypes. We detected increased levels of charged lipids (SQDG and PG) in the Lut overaccumulating plants compared with other examined genotypes, in particular in EL conditions (Supplemental Figure S6).

### The efficiency of photosynthetic apparatus is mostly affected in Lut-deficient *ccr1-1* plants

**Figure 9:**
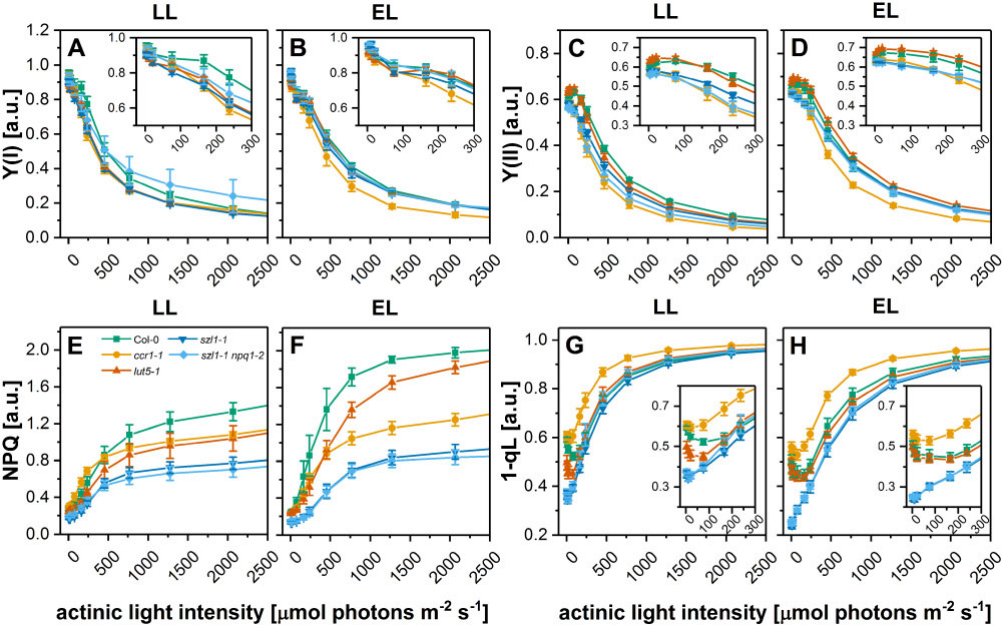
Efficiency of photosynthetic light reactions of Columbia-0 (Col-0) and carotenoid-deficient Arabidopsis (*A. thaliana*) plants grown in LL and adapted to EL conditions. A–H, Changes of photosynthetic parameters values. A, B, Effective quantum yield of photosystem I (PSI) (Y(I)). C, D, Effective quantum yield of PSI Y(II). E, F Nonphotochemical quenching (NPQ). G, H, Excitation pressure (1-qL) measured under increasing light intensities. Data are mean values ± SE from at least three independent experiments.

To reveal the influence of the altered carotenoid composition on the efficiency of the photosynthetic apparatus we measured the photochemistry of both photosystems in all examined plants (Figure 9, Supplemental Figure S7). We recorded induction curves at high light intensities (475 *µ*mol photons m^−2^ s^−1^) which revealed faster and more clear on-light relaxation of NPQ in both Lut overaccumulating mutants compared with other examined genotypes (Supplemental Figure S7A–E). In the case of the *ccr1-1* mutant, the induction curve course was significantly different from other examined plants and did not indicate any on-light NPQ relaxation (Supplemental Figure S7B). The analysis of photosynthetic parameters during illumination with exponentially increasing light intensities showed that the Y(I) parameter decreased faster in all carotenoid-deficient mutants compared with wt plants in the initial phase of illumination (up to 300 *µ*mol photons m^−2^ s^−1^; Figure 9A). In EL conditions, the decrease in Y(I) parameter was faster only in the *ccr1-1* mutant compared with other examined plants in the whole range of applied light intensities (Figure 9B). The decrease of Y(II) was faster in LL and EL in *ccr1-1* and LL only in *szl1-1* and *szl1-1npq1-2* plants (Figure 9, C and D). Changes in the NPQ parameter revealed that Col-0 plants had better capability to dissipate energy in high light intensities compared with all carotenoid-deficient mutants, especially *szl1-1* and *szl1-1npq1-2* (Figure 9, E and F). The measurement of 1-qL parameter showed that the highest excitation pressure was present in the LL *ccr1-1* plant (Figure 9G). Similar course of the 1-qL light curve was observed also for EL plants with a general decrease in the excitation pressure values due to better adaptation of EL plants to higher light intensities (Figure 9H).

## Discussion

### Linking structural and functional level of grana formation—enigmatic role of carotenoids

Internal membrane network in chloroplasts of higher plants is built by the intricate grana and stroma thylakoid system, which generally adopts pitch-balanced helical arrangement (Bussi et al., 2019). Reasons for the evolution of grana remains vague (Anderson et al., 2008; Puthiyaveetil et al., 2016), but the photochemical and nanophotonic benefits of such morphology are indubitable (Iwai et al., 2016; Capretti et al., 2019) and especially relevant in the context of plants with altered composition of accessory pigments, i.e., carotenoids.

Recent studies on precursor thylakoid membranes, i.e. membranes of etioplast prolamellar bodies (PLBs), show that changes in the lipid phase carotenoid composition can modulate PLB structure significantly—from local aberrations in bicontinuous structure (Bykowski et al., 2020) to complete arrest of PLB folding (Cazzonelli et al., 2020). These studies, performed on membranes with similar to thylakoids lipid composition, however, lacking carotenoid-binding protein complexes, bring attention to the potential direct and indirect role of carotenoid components in the formation of particularly formed grana entities and the organization of the whole thylakoid network.

### Light-affected spatial dynamics of the thylakoid network and stable grana structure in *ccr1-1* mutant

Results obtained for the *ccr1-1* plants showing no changes in grana structure and CP complex organization (Figures 2, 6, 7) are in line with the previous studies on this mutant, which indicated reduction and delay in the NPQ mechanism and a slight decrease in the Chl content, with no significant changes in the general chloroplast morphology compared to wt plants (Cazzonelli et al., 2009). However, our spatial structural analyses revealed that the sphericity of the thylakoid network in the *ccr1-1* mutant decreased after exposition to EL conditions with no light-dependent changes in grana sizes and SRD values measured from the TEM data (Figures 3, 5). Therefore, flattening of the thylakoid network in the *ccr1-1* mutant is most probably related to the decrease in the distances between neighboring grana in the vertical plane leading to the thylakoid network compression. Such changes in the distances between grana entities were recently postulated to be one of the mechanisms regulating the photosynthesis capacity (Capretti et al., 2019).

### Increased grana stacking related to larger availability of thylakoid components in *the lut5-1* mutant—complex regulation of β-ring hydroxylation

In *lut5-1* plants, we observed higher grana stacks compared to other examined genotypes (Figure 2), which correspond to the reported structure of *lut-2—*another mutant in Lut biosynthesis pathway (Dall’Osto et al., 2007). Higher total carotenoid and Chl level per dry weight and markedly increased level of LHCII proteins in the *lut5-1* mutant (Figure 1, Supplemental Figure S4) compared with Col-0 might partially explain larger grana stacks of *lut5-1* due to the higher availability of thylakoid building components. In previous studies, it has been reported that the *lut5* mutant is light sensitive upon transfer to high light conditions (1000–1800 *µ*mol photons m^−2^ s^−1^; Fiore et al., 2006; Kim and DellaPenna, 2006; Kim et al., 2009; Beisel et al., 2010). However, in the range of light intensities used in our experiments, we did not notice significant changes in the photosynthetic efficiency in this mutant between LL and EL conditions. High Car turnover rate in plants growing in moderate light conditions has been previously confirmed (Beisel et al., 2010) and four genes coding β-ring hydroxylases were identified in Arabidopsis (Fiore et al., 2006; Kim et al., 2009). Registered light-dependent dynamic change in α/β-Car ratio in *lut5-1* (Supplemental Table S1), suggests next level of carotenoid biosynthesis pathway regulation via activation of additional enzymes performing β-ring hydroxylation (BCH1, BCH2, CYP97C1) in plants adapted to EL conditions.

### Decrease in lipid-phase fluidity driven by the Lut/Car ratio and its complex role in membrane folding

In both Lut overaccumulating mutants, we registered a decrease in the size of grana stacks (Figures 2, 3). Such differences in grana sizes are in line with the 3D shape of the thylakoid network exhibiting lower values of the area/volume ratio in plants with small grana stack, and therefore giving a direct evidence *in vivo* of significant changes in the grana folding between the examined genotypes (Figure 5).

Modulation of the membrane fluidity has been attributed directly to the composition of lipid-phase located carotenoids. In particular, the increased Lut/Car ratio was associated with thylakoid membrane rigidification due to the distinct orientation of Lut and Car in a lipid bilayer (Gruszecki and Strzałka, 2005). In *szl1-1* and *szl1-1npq1-2* plants, we observed overaccumulation of Lut, no concomitant increase in Lut-binding LHC proteins (Figures 7–8, Supplemental Figure S4), and no simultaneous reduction in the Car level compared with other examined genotypes (Figure 1). These results together with Laurdan fluorescence measurements (Figure 6) point to further decrease in the fluidity of the thylakoid matrix of the *szl1-1* and *szl1-1npq1-2* plants associated with the direct Lut accumulation in the lipid bilayer.

Moreover, the Lut-dependent increase in membrane rigidity (Figure 6) might also be responsible for the formation of low-mass PSII supercomplexes in the *szl1-1* and *szl1-1npq1-2* plants (Figure 7) due to the reduced mobility of protein complexes in a less fluid environment (Mullineaux, 2008). It also explains limited transfer of excitation from antennae to PSII core complexes registered in these mutants (Supplemental Figure S3). Previous studies showed that increased membrane rigidity might result in a reversible binding of soluble proteins with the thylakoid surface (Mazur et al., 2018). Similarly, in the case of the *szl1-1* and *szl1-1npq1-2* plants, we observed binding of Rubisco large subunit to thylakoids (Figure 8). In chloroplast stroma, Rubisco is organized in the form of a functional octamer and might also form larger megadalton complexes with other Calvin–Benson–Bassham cycle enzymes (Olinares et al., 2010). The presence of Rubisco octamer in the vicinity of rigid thylakoids could provide a steric hindrance for the process of membrane folding visible as hampered grana formation.

We further investigated whether carotenoid-dependent decrease in the thylakoid fluidity in the *szl1-1* and *szl1-1npq1-2* plants (Figure 6) is not compensated by the increase in the MGDG/DGDG ratio also strongly related to physical membrane properties (Demé et al., 2014). In the TLC analysis, we observed only an increase in the charged lipids presence (SQDG and PG) in both Lut overaccumulating plants (Supplemental Figure S6). The majority of grana thylakoid lipids are positioned in the lipid–protein interface (Páli et al., 2003). Interestingly, head groups of charged lipids bound with the PSII–LHCII supercomplex are positioned exclusively on the stromal side of the thylakoid membrane (Sheng et al., 2018). Thus, their increased amount markedly influences the amount of negative charge on the PSII–LHCII stromal surface and together with increased phosphorylation of LHCII antennae (Supplemental Figure S5) also contribute to hampered grana folding in the *szl1-1* and *szl1-1npq1-2* plants.

The obstacle in grana folding provided directly by rigid membranes has not been recognized yet. Probably, the above described significant changes in lipid–pigment–protein interactions provided by, but also resulting from, the membrane stiffness, together with slowed dynamics of the thylakoid system (Yamamoto, 2016), are the main factors leading to hampered grana formation in the Lut overaccumulating plants.

### Carotenoid-dependent changes in phosphorylation profile of thylakoid proteins—possible role in the retardation of grana folding

The STATE TRANSITION 8 (STN8) kinase plays a primary role in the phosphorylation of PSII core proteins (D1, D2, CP43, PsbH); however, the mechanism of STN8 activation is still unclear (Rochaix, 2013). In the case of *szl1-1* and *szl1-1npq1-2* plants, lack of STN8-dependent phosphorylation (Supplemental Figure S5) is not related to altered level of STN8 kinase (Supplemental Figure S5B) nor availability of PSII core phosphorylation sites (Supplemental Figure S8). Although Lut overaccumulating plants have altered Car composition of PSII (Cazzaniga et al., 2012), pigment binding sites in PSII core complex are spatially distant from its phosphorylation sites. Regarding the STATE TRANSITION 7 (STN7) kinase, the mechanism of its activation is relatively well known and dependent on plastoquinone reduction (Tikkanen and Aro, 2012). Therefore, it is directly related to the efficiency of photosynthesis light reactions. Considering such results and high similarity of STN7 and STN8 kinases (Wunder et al., 2013), we suspect that the inhibition of STN8 activity might be related to the decrease in NPQ values resulting in a substantial reduction in the excitonic pressure registered in the *szl1-1* and *szl1-1npq1-2* plants (Figure 9).

Moreover, an increase in the presence of PSII supercomplexes (those of low-weight particularly), registered in the BN-electrophoretic pattern of the *szl1-1* and *szl1-1npq1-2* plants (Figure 7), is consistent with the results obtained for the *stn8* mutant (Wunder et al., 2013). A rise in the abundance of PSII supercomplexes has been associated with a higher lateral stability of supercomplexes when the PSII core is not phosphorylated, and therefore the negative charge of PSII core is decreased (Wunder et al., 2013). Finally, the accumulation of PSII supercomplexes leads to a decrease in the fluidity of the thylakoid membranes (Pesaresi et al., 2011). Thus, the reduced amount of the PSII-located Car eventually resulted in a significant change in the organization of the PSII supercomplexes leading to an increase in the rigidity of thylakoids in the *szl1-1* and *szl1-1npq1-2* mutants.

Additionally, we confirmed the presence of the PSI–LHCI–LHCII complex together with an increased phosphorylation of Lhcb1 and Lhc2 proteins in the *szl1-1* and *szl1-1npq1-2* plants only (Figure 7, Supplemental Figure S5). It was previously established that the s*zl1-1* mutant has an enhanced sensitivity to photoinhibition, mainly due to high susceptibility of Car-depleted PSI to light stress. In the optimal growth conditions, the *szl1-1* plants exhibit a decreased LHCI complex level and capacity for Chl fluorescence quenching (Cazzaniga et al., 2012). Therefore, the presence of PSI–LHCI–LHCII complex in both examined light conditions in the *szl1-1* and *szl1-1npq1-2* plants together with an increased amount of grana end-membranes (Supplemental Figure S2) can be considered as a compensation mechanism, which replaces missing LHCI antennae and also increases the PSI efficiency in these mutants (Marutani et al., 2017).

### Limited effect of carotenoid deficits on granum diameter

Changes in the diameter of grana stacks were observed in plants with mutations in different thylakoid components e.g. galactolipid-deficient plants, oe*CURT1A* line, and *stn7stn8* double mutant (Fristedt et al., 2009; Armbruster et al., 2013; Mazur et al., 2019). Almost no registered change in grana diameter (Figure 3) together with a stable level of CURT1A protein between Col-0 and examined carotenoid-deficient mutants (Figure 8) in both light conditions indicate a limited role of the carotenoid component in the tuning of grana width.

### Potential role of plastoglobules in δ- and γ-carotene accumulation

The carotenoid presence was detected in the chloroplast plastoglobules and it is known that their biosynthesis pathway is partially executed in this membrane compartment (van Wijk and Kessler, 2017). We registered increased plastoglobule sizes (Figure 3) in mutants with altered composition of Cars (Figure 1). Both δ-Car and γ-Car, registered in *szl1-1* and *szl1-1npq1-2* plants with retarded activity of the LYC-B enzyme (Supplemental Figure S1), are direct precursors of α-Car and β-Car, respectively, and do not accumulate in chloroplasts of higher plants (Kim et al., 2009). The localization of LYC-B and its significant abundance has been previously confirmed in plastoglobules of chromoplasts (Ytterberg et al., 2006). Moreover, the analysis of the LYC-B protein structure revealed lack of transmembrane domains pointing to its association with the plastoglobule fraction (van Wijk and Kessler, 2017). Previous studies on the carotenoid composition of *szl1-1* CP complexes confirmed the presence of β-Car and α-Car bound to PSs cores with no appearance of δ-Car and γ-Car (Cazzaniga et al., 2012). Therefore, most probably, LYC-B substrates (δ-Car and γ-Car) are located directly in large plastoglobules of the *szl1-1* and *szl1-1npq1-*2.

## Conclusions

**Figure 10:**
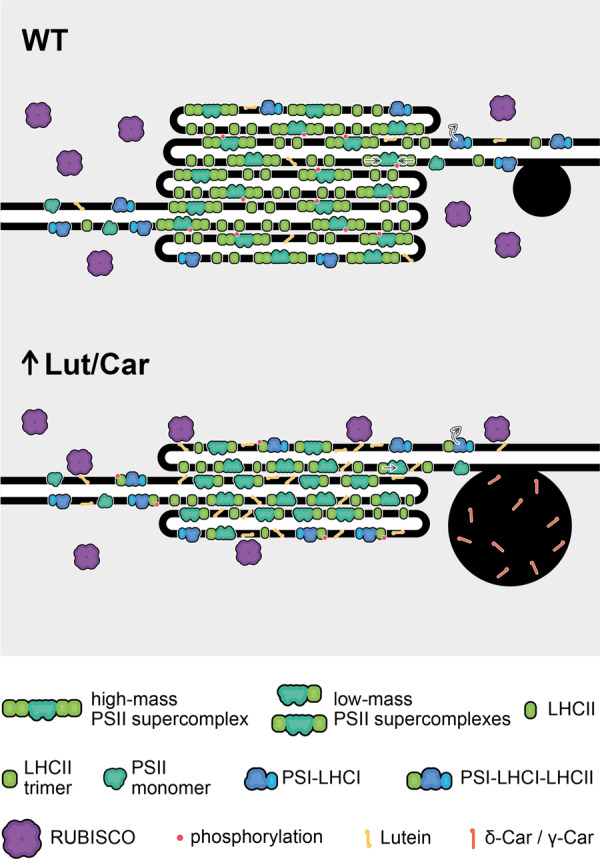
Scheme summarizing the influence of increased lutein (Lut) to carotene (Car) ratio on the structure and supramolecular organization of the thylakoid membranes. Carotenoid-dependent retardation in grana folding is related to the decrease in thylakoid fluidity. This is caused by the synergistic effect of overaccumulation of lutein in thylakoid matrix and increased level of nonphosphorylated low-mass PSII supercomplexes due to the decreased level of β-carotene bound with PSII core complex. Decreased thylakoid fluidity results in: (i) reversible binding of Rubisco to the thylakoid surface providing steric hindrance for further membrane folding, (ii) retardation in the PSII core-antennae spectral connectivity, which leads to a limited transfer of excitation energy to the PSII reaction center. Another factor that might further negatively influence membrane folding is an increased light harvesting complex II (LHCII) protein phosphorylation in plants with increased Lut/Car ratio. Highly phosphorylated LHCII pool at least partially forming PSI–LHCI–LHCII supercomplexes, which might compensate a decreased level of light harvesting complex I (LHCI) antennae and influence the PSI efficiency. Interestingly, such plants accumulate additionally δ-Car and γ-Car, which are not typically found in thylakoid membranes of higher plants; however, their presence in chromoplasts was confirmed earlier. We speculate, that δ-Car and γ-Car are mostly present in significantly enlarged plastoglobules of the lutein overaccumulating mutants and do not directly influence thylakoid network structure.

This study provides an important contribution to our understanding of a link between carotenoid composition and the thylakoid network structure, folding of grana entities in particular. Our results point to essential role of the Lut/Car balance in the grana structure formation. The scheme summarizing changes in the carotenoid-dependent supramolecular organization of the thylakoid membrane leading to substantial reduction of grana folding in the *szl1-1* and *szl1-1npq1-2* plants is presented in Figure 10. The synergistic effect of Lut overaccumulation in a lipid bilayer and β-Car deficiency in the PSII core complex leads to a decrease in the thylakoid membrane fluidity caused by direct changes in the thylakoid matrix as well as changes in the supramolecular organization of photosynthetic complexes and their interactions. Stiffer thylakoids finally resulted in hampered process of grana folding in the *szl1-1* and *szl1-1npq1-2* plants. Apart from the direct role of carotenoids in light-harvesting and supercomplexes formation, less defined grana structures, with decreased efficiency of light absorption, might also be the reason of reduced fitness of the *szl1-1* and *szl1-1npq1-2* plants reflected by decreased F_V_/F_M_ values. Such an approach links directly ultrastructural and molecular levels of carotenoid significance in the establishment of photosynthetic efficiency. In a broader context, this study concerns the interplay between the structure and composition of the thylakoids, as such, regards deciphering the key membrane components playing a regulatory role in the self-assembly mechanism of the thylakoid network folding.

## Materials and methods

### Plant material and growth conditions

Seeds of *A. thaliana* mutants *ccr1-1* (N68151, *sdg8*; Park et al., 2002), *lut5-1* (N616660, SALK_116660; Kim and DellaPenna, 2006), *szl1-1* (N66022; Li et al., 2009), *szl1-1npq1-2* (N66023; Li et al., 2009), and ecotype Col-0 (N1092, control) were obtained from The European Arabidopsis Stock Center. Plants were grown in peat pellets for 10 weeks in 8 h photoperiod at 22°C/18°C (day/night) at PAR 70 *µ*mol photons m^−2^ s^−1^. Plants used for EL experiments were grown in the same conditions for the first 9 weeks and then at the photoperiod at PAR 120 *µ*mol photons m^−2^ s^−1^ for the last, 10th week of growth. All samples were collected at the beginning of the day period—during the first hour of light.

### Extraction of nonpolar lipids and analysis of carotenoids

Chl and carotenoid concentrations were determined spectrophotometrically after extraction with 80% (v/v) acetone (Mazur et al., 2016). For liquid chromatography analysis, nonpolar lipids, including carotenoids, were extracted using method given in Szalonek et al. (2015). Extracted pigments were separated using the Shimadzu Prominence HPLC System with PDA detector using Atlantis C-18 3-*µ*m 30 × 150 mm Waters column with Supelguard™ Ascentis™ C18 guard column (5 μm, 4.0 × 20 mm). Elution of pigment samples was performed using ethyl acetate gradient in acetonitrile: water: triethylamine 9:1:0.01 (v/v) at 1 mL min^−1^ for 37 min. Additionally, extracted pigments were analyzed using ACQUITY UPLC HSS T3 system on 1.8-*μ*m 1.0 × 150 mm column via the method described in Skupień et al. (2017). For the identification of γ-Car and δ-Car the UPLC unit was connected with Synapt G2 HDMS mass spectrometer (Waters). The mass spectra were recorded in positive electrospray ionization mode in a mass range from m/z 200–1,000 in low-energy TOF MS mode.

### Chlorophyll *a* fluorescence and P700 measurements *in vivo*

Arabidopsis plants were dark-adapted for 30 min and subsequently Chl-fluorescence images were recorded with the help of the Imaging-PAM Chl fluorescence system (Heinz Walz GmbH) under actinic light illumination (475-*μ*mol photons m^−2^ s^−1^) and dark recovery. Simultaneous Chl *a* fluorescence and P700 measurements on 30 min dark-adapted Arabidopsis leaves were carried out using the Dual-PAM 100 fluorometer (Heinz Walz GmbH) according to Mazur et al. (2016).

### Transmission electron microscopy

Samples for TEM were prepared according to Mazur et al. (2016). TEM images were collected with the help of the JEM 1400 microscope (JEOL) equipped with Morada G2 (EMSIS GmbH) CCD camera, Poland. Different ultrastructural features of grana were calculated with the help of iTEM software (Olympus).

### CLSM and 3D reconstruction *in vivo* of mesophyll chloroplasts

Samples of single mesophyll cells for chloroplast *in vivo* 3D analysis in CLSM were prepared and analyzed as described before (Mazur et al., 2019) using Nikon A1 MP microscope equipped with an Plan Apo TIRF x100 oil differential interference contrast H (numerical aperture = 1.45) objective lens. An excitation beam of 561.2 nm was obtained from a laser working at 4% of the nominal power. Fluorescence emission was recorded in the range of 662–737 nm, the confocal aperture was set at 1 Airy unit. Stacks of 512 × 512-pixel images were taken with the *z*-axis step set at 60 nm and detector parameters set to avoid pixel oversaturation (gain = 85, offset =-40). Structural features of 3D models were calculated using the MeasurementPro package of Imaris 8.4.2.

### Spectroscopy measurements

Circular dichroism (CD), low-temperature (77 K), and RT fluorescence were performed on thylakoid membrane fraction isolated as described in Garstka et al. (2007) and diluted to Chl concentration of 10 μg mL^−1^. For CD measurements we followed the procedure described in Mazur et al. (2019). The Chl emission spectra were recorded using modified Shimadzu RF-5301PC as described in Mazur et al. (2019). The Chl fluorescence excitation spectra were recorded using FS5 Spectrofluorometer (Edinburgh Instruments) equipped with 77 K Optistat DN2 cryostat (Oxford Instruments). For membrane fluidity measurements, isolated thylakoids were diluted to Chl concentration of 2.5 μg mL^−1^ in 20-mM Hepes buffer (pH 7.5) containing 330-mM sorbitol. After 10 min at 25°C, sample was incubated with 1-*µ*M laurdan for 30 min at 25°C. Steady-state fluorescence emission spectra were recorded (Shimadzu RF-5301PC) at 25°C in the range of 400–600 nm after excitation at 390 nm; excitation and emission slits were set to 10 and 5 nm, respectively. Generalized polarization values were calculated according to the formula presented in Figure 6G.

### Protein electrophoretic analyses

Samples for Blue native PAGE (BN-PAGE) were prepared according to Mazur et al. (2019) with slight modifications. Thylakoid samples containing 8.3 *µ*g of Chl were loaded into gel wells. Electrophoresis was carried out using 4%–16% (w/v)-gradient acrylamide gels according to the manufacturer protocol (Invitrogen). BN-PAGE lanes were denatured in 125-mM Tris-HCl with pH 6.8, 5-M urea, 10% (v/v) glycerol, 5% (w/v) sodium dodecyl sulfate (SDS), and 5% (v/v) β-mercaptoethanol for 30 min at 65°C. Water-washed strips were placed onto gels and sealed with 1% (w/v) agarose. 2D electrophoresis was performed and gels were stained as described in Mazur et al. (2019).

Thylakoid samples containing 1 *µ*g of Chl were loaded onto gels and separated by the standard SDS-PAGE electrophoresis protocol, transferred, and detected on PVDF membranes by antibodies (Agrisera) against selected proteins. Visualization was obtained using anti-rabbit HRP-conjugate and ECL Detection System (BioRad). Moreover, additional SDS-PAGE thylakoid separations were performed on large 14%–20% (w/v) polyacrylamide gels.

### Thin layer chromatography

Total lipid extracts were obtained as described previously (Skupień et al., 2017) and separated by TLC. Lipid extracts containing 150 *µ*g of Chl were deposited on HPTLC Silica gel 60 plates (Supelco) and separated in developing chamber containing acetone: toluene: water 91:30:7.5 (v/v/v) mobile phase. Developed plates were dried, sprayed with 50% (v/v) H_2_SO_4_, and heated at 100°C for 5–10 min. Lipids were identified by comparison with standards developed in the same conditions.

### Statistical analysis

The statistical significance of differences between results was determined by one-way ANOVA with post hoc Tukey test at *p *≤* *0.05. For the number of repetitions of specific experiments as well as number of calculated structural features (*n*), see Figure and Table captions.

## Accession numbers

GenBank/EMBL accession numbers are *AT1G06820* (*CRTISO*), *AT1G31800* (*LUT5*), *AT3G10230* (*SZL1*), *AT1G08550* (*NPQ1*), *ATCG00020* (*PSBA*), *ATCG00280* (*PSBC*), *AT2G05100* (*LHCB2.1*), *AT2G05070* (*LHCB2.2*), *AT3G27690* (*LHCB2.3*), *ATCG00490* (*RBCL*), *AT4G01150* (*CURT1A*), *AT5G01920* (*STN8*).

## Supplemental data

The following materials are available in the online version of this article.

**Supplemental Figure S1**. Identification of nontypical peaks present in chromatogram of carotenoid extract of *szl1-1* plants.

**Supplemental Figure S2**. Quantitative changes in additional ultrastructural parameters of the thylakoid network.

**Supplemental Figure S3**. Low-temperature (77 K) chlorophyll fluorescence excitation spectra of thylakoids.

**Supplemental Figure S4**. Quantitative analysis of selected photosynthetic complexes and protein abundance in thylakoids.

**Supplemental Figure S5**. Phosphoprotein staining and STATE TRANSITION (STN8) level in SDS-PAGE separated thylakoid samples.

**Supplemental Figure S6**. TLC of total leaf lipid extracts.

**Supplemental Figure S7**. NPQ measurements.

**Supplemental Figure S8**. PSII protein models showing β-carotene localization and STATE TRANSITION 8 (STN8) kinase phosphorylation sites.

**Supplemental Table S1**. Carotenoid and antioxidant component ratios.

## Supplementary Material

kiaa009_Supplementary_Data

## Notes

Ł.K., M.B., and R.M. designed experiments; A.M. and Ł.K. provided conception of the paper; Ł.K. coordinated the project; M.B., R.M., J.W., Ł.K., and M.G. performed experiments; M.B., R.M., J.W., Ł.K., M.G., and S.S. processed data; Ł.K., A.M., and R.M. wrote the article with a contribution of J.W.

The author responsible for distribution of materials integral to the findings presented in this article in accordance with the policy described in the Instructions for Authors (https://academic.oup.com/plphys) is: Ł.K. (lucja.kowalewska@uw.edu.pl).
