# Supplementary material for: Too rigid to fold: Carotenoid-dependent decrease in thylakoid fluidity hampers the formation of chloroplast grana
Source: Plant Physiol. 2020 Nov 28;185(1):210–27. doi: 10.1093/plphys/kiaa009 (PMC8133577; doi:10.1093/plphys/kiaa009)
Supplement: kiaa009_Supplementary_Data [file kiaa009_supplementary_data.zip › kiaa009-suppl_data/pp.01286.2020-s01.pdf]

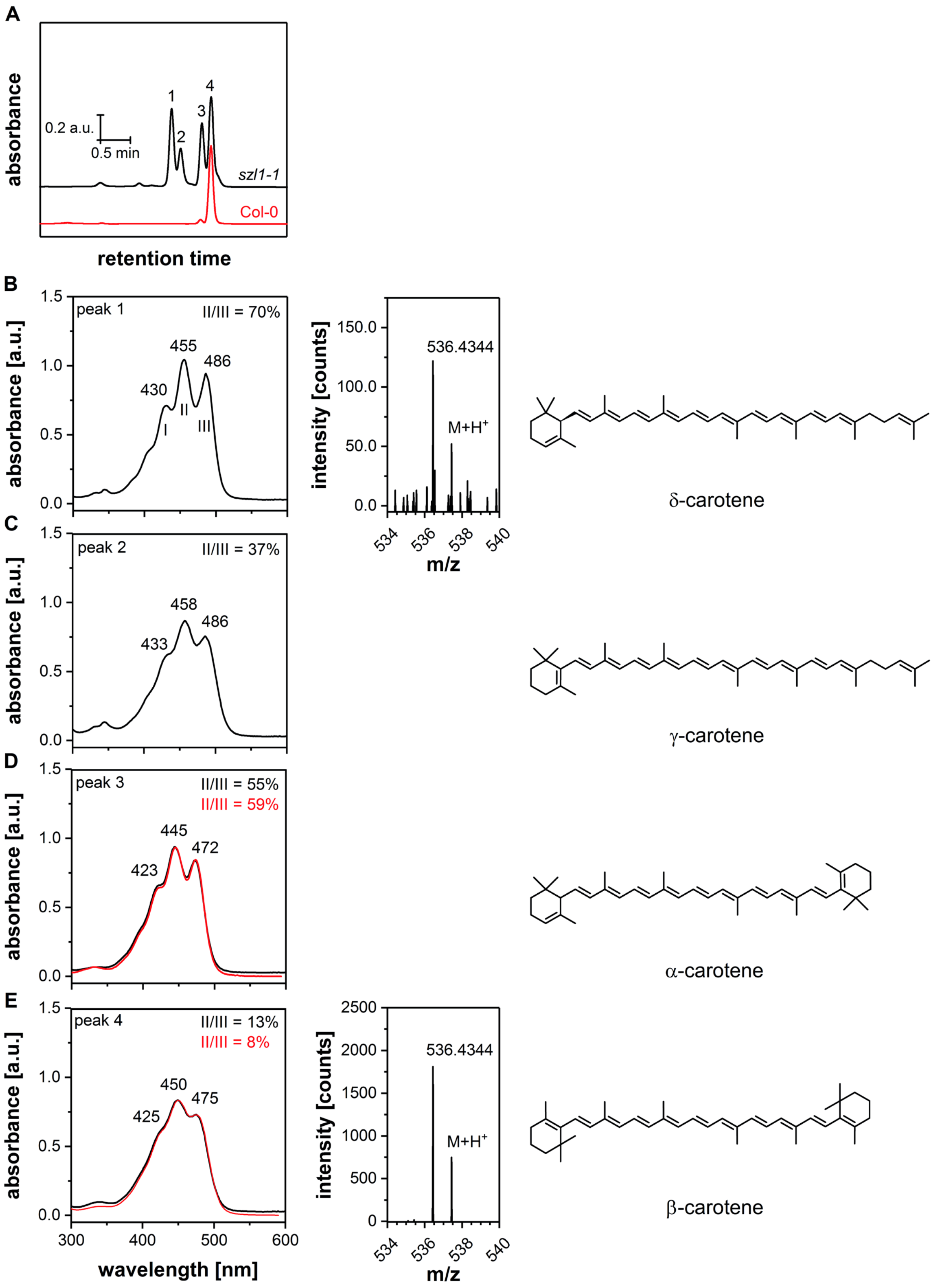

Supplemental Figure S1

Identification of non-typical peaks present in chromatogram of carotenoid extract of *szl1-1* plants.

A – UPLC chromatogram in the region of carotene retention times; B-E – absorption spectra of respective chromatogram peaks with corresponding mass-spectra identified for δ and β carotene (Alagoz et al. 2020); note that the presence of peak 1 and 2 was confirmed in LL and EL conditions for both *szl1-1* and *szl1-1npq1-2* extracts.

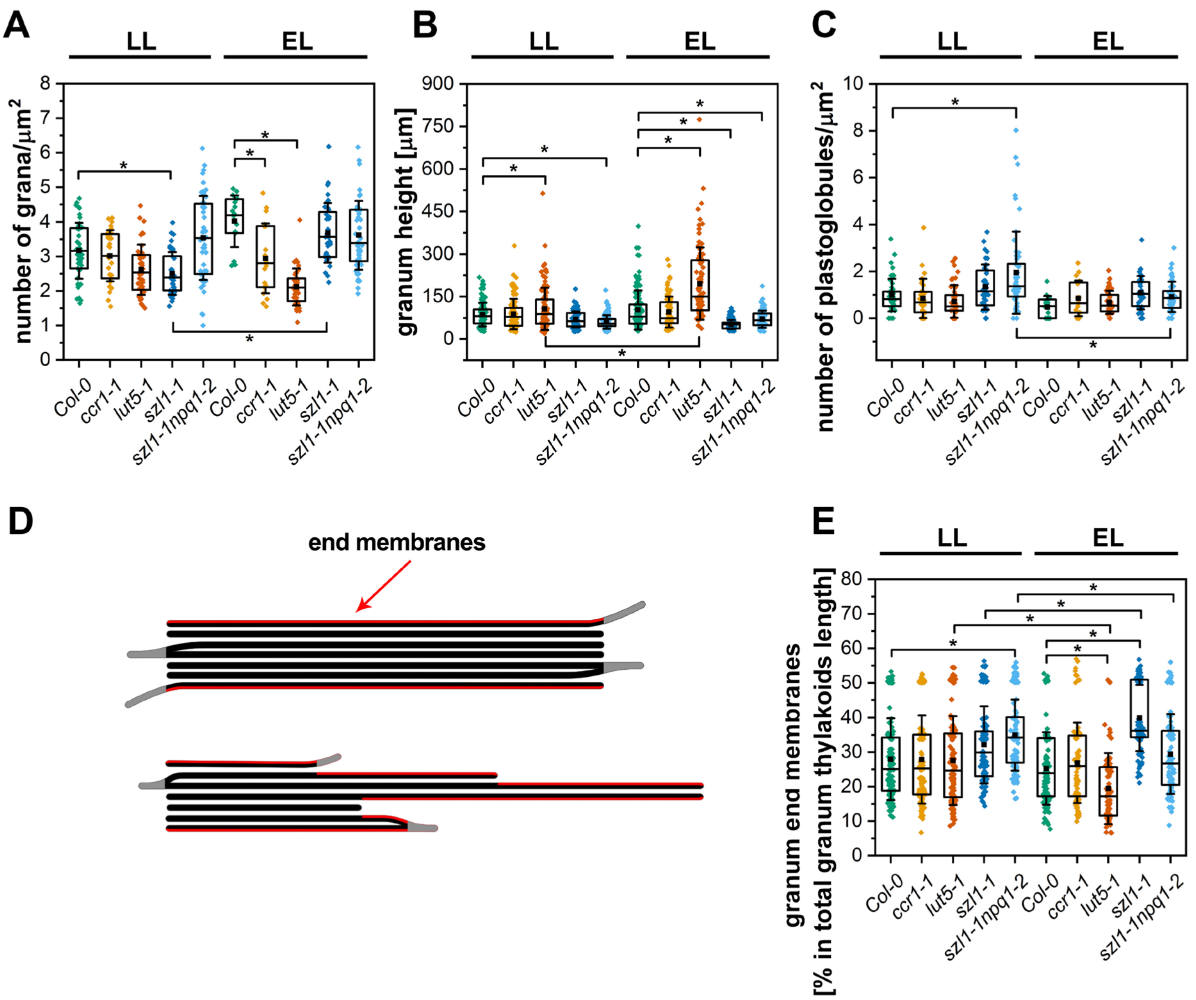

Supplemental Figure S2

Quantitative changes in additional ultrastructural parameters of the thylakoid network.

Parameters were calculated and measured from electron micrographs of Columbia-0 (Col-0) and carotenoid-deficient Arabidopsis plants grown in low light (LL) and adapted to excessive light conditions (EL). A – number of grana stacks per μm^2 of the chloroplast cross-section; B – granum height; C – number of plastoglobules per μm^2 of the chloroplast cross-section; D – scheme showing localization and length of end-membranes in grana stacks with different grana lateral irregularity; E – percentage share of granum end-membrane length in the total granum thylakoid length; the bottom and top of each box represent 25 and 75 percentile respectively. The whiskers denotes standard deviation (SD) and every point visible on the boxes represent individual measurement; pairs of results marked with asterisk differ significantly at p = 0.05 (one-way ANOVA with post-hoc Tukey test; n = 90 – 144); only differences between mutants and Col-0 in respective light conditions and for particular genotypes in both light conditions are marked.

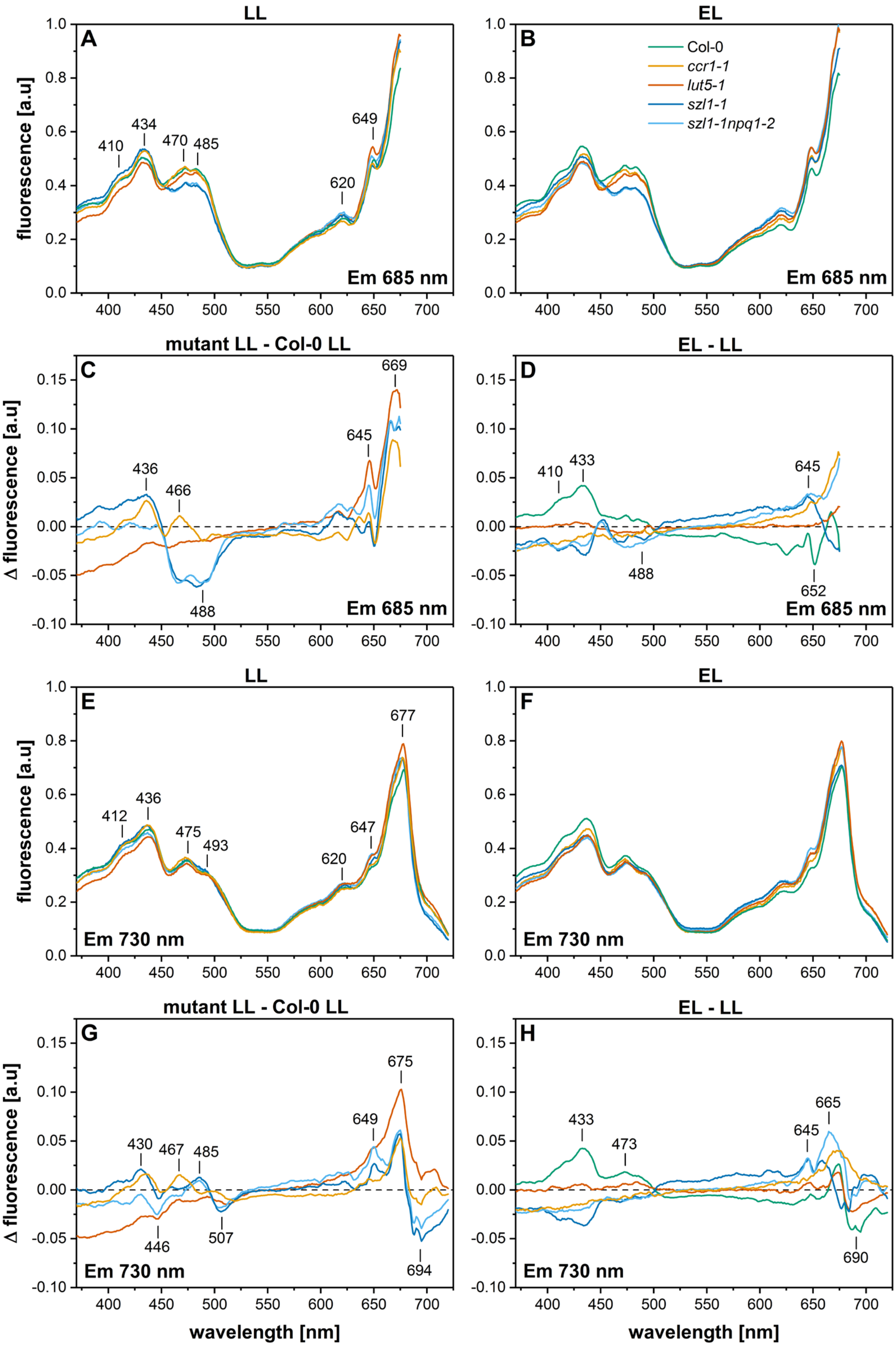

Supplemental Figure S3

Low-temperature (77 K) chlorophyll fluorescence excitation spectra of thylakoids.

Spectra were recorded for Columbia-0 (Col-0) and carotenoid-deficient Arabidopsis plants grown in low light (LL) and adapted to excessive light conditions (EL). A, B, E, F – excitation–spectra obtained from emission at 685 nm (A-B) and 730 nm (E-F) and normalized to equal area under the curve; C, D, G, H – fluorescence excitation difference spectra mutant-minus-Col-0 in LL conditions (C, G) and EL-LL for all genotypes (D, H); excitation and emission slits – 3 nm; scan ranges – 350-680 and 350-725 nm. Spectra were recorded through the LP610 emission filter and were corrected for the xenon lamp intensity and photomultiplier sensitivity.

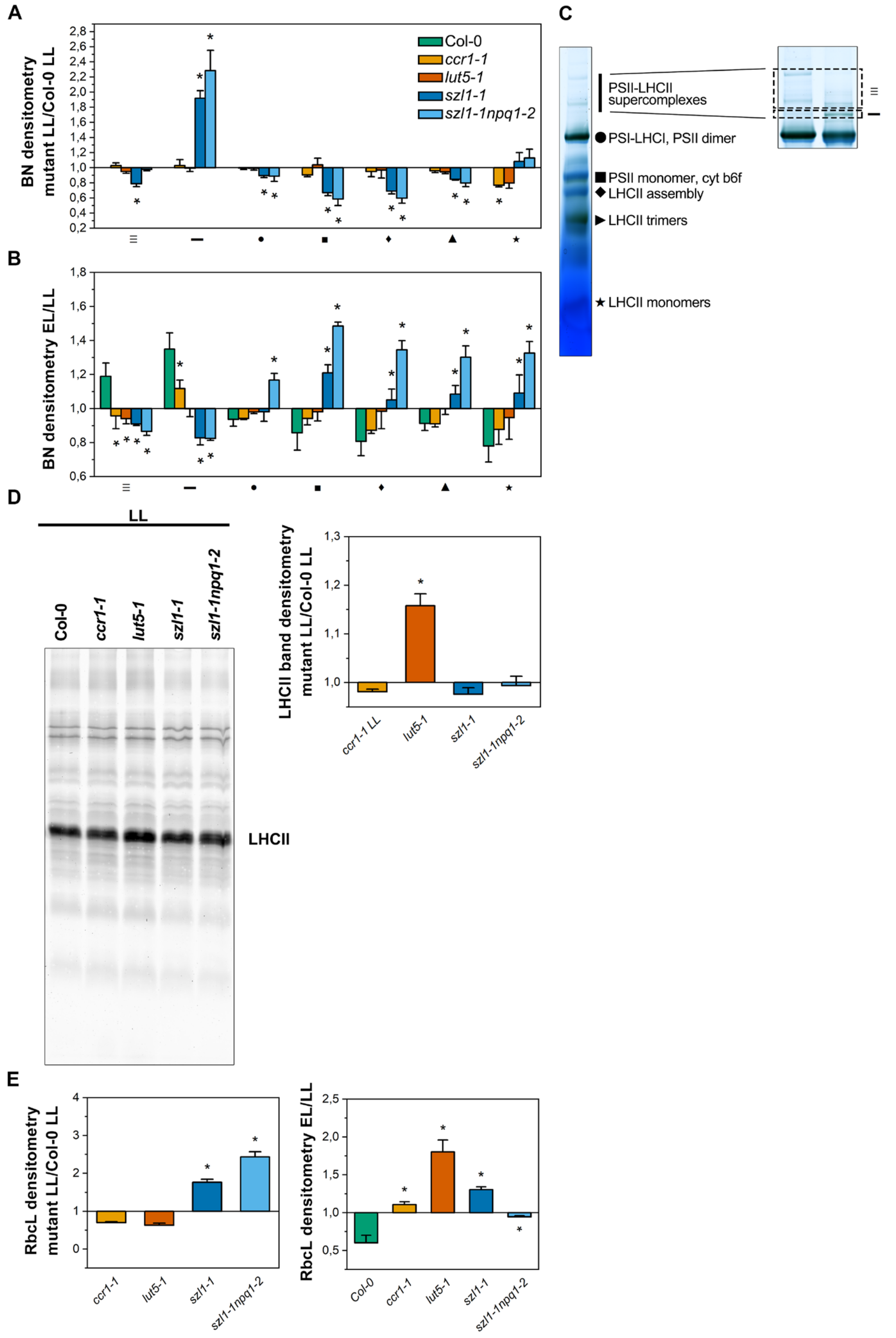

Supplemental Figure S4

Quantitative analysis of selected photosynthetic complexes and protein abundance in thylakoids.

Proteins were analyzed for Columbia-0 (Col-0) and carotenoid-deficient Arabidopsis plants grown in low light (LL) and adapted to excessive light conditions (EL). A-C – densitometry of bands corresponding to particular photosynthetic complexes separated via BN-electrophoresis for mutant LL samples in respect to Col-0 LL (A) and EL/LL for all genotypes separately (B), note that the legend for geometrical shapes symbolizing specific complexes is given in the panel C (photosystem I (PSI), photosystem II (PSII), light harvesting complex I (LHCI), light harvesting complex II (LHCII); D – SDS page separation of thylakoid proteins with corresponding densitometry analysis showing a significant increase in the abundance of LHCII in the *lut5-1* plants in respect to Col-0; E – densitometry of RbcL immunodetection, calculations as for A and B; data are mean values ± SD from 3 independent experiments; results marked with asterisk differ significantly at p = 0.05 (one-way ANOVA with post-hoc Tukey test) from Col-0 in LL conditions or between LL and EL conditions for particular genotype. The abundance of selected photosynthetic complexes and proteins was determined densitometrically using the Image-Lab 6.0.1 software (Bio-Rad).

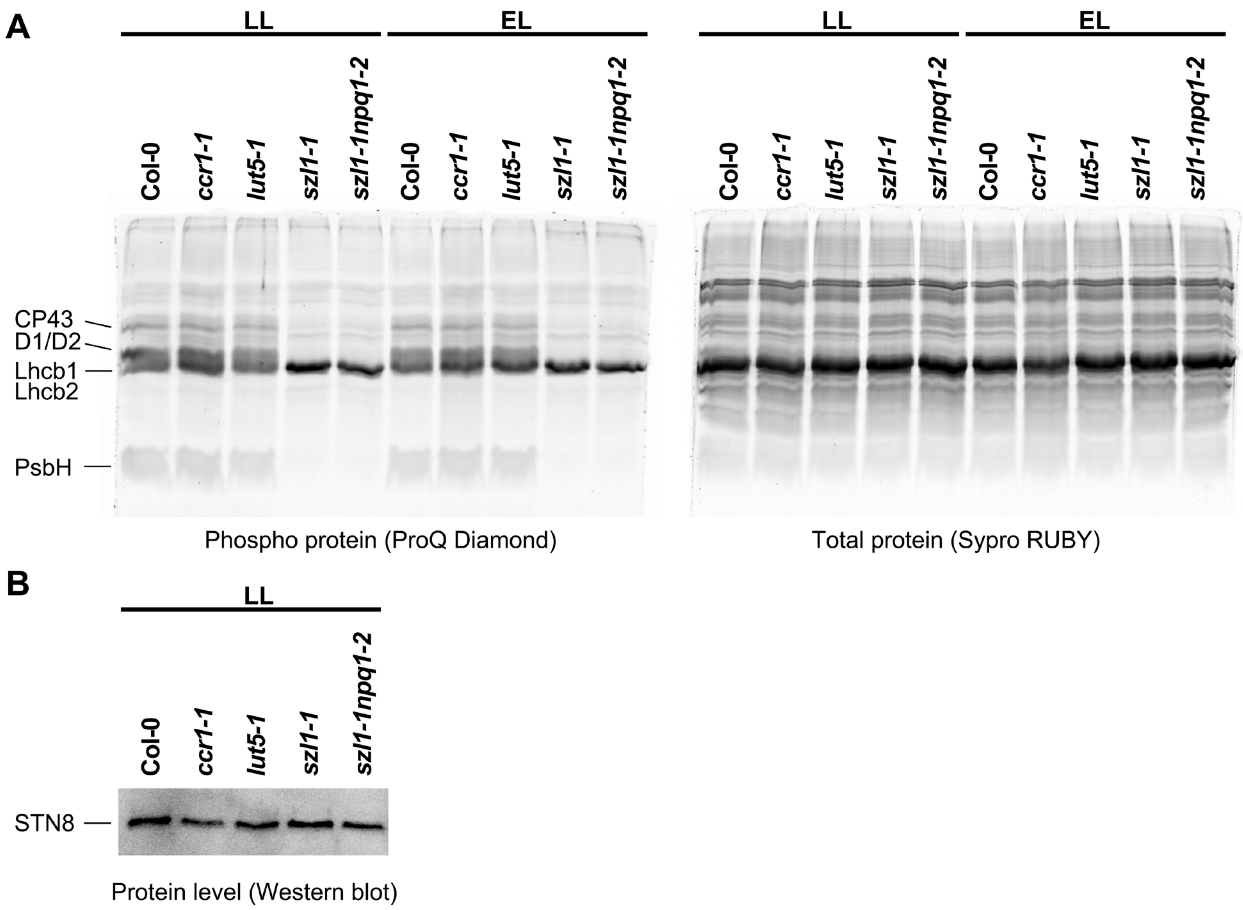

Supplemental Figure S5

Phosphoprotein staining and STATE TRANSITION 8 (STN8) level in SDS-PAGE separated thylakoid samples.

Analysis was performed for Columbia-0 (Col-0) and carotenoid-deficient Arabidopsis plants grown in low light (LL) and adapted to excessive light conditions (EL). A – the level of thylakoid protein phosphorylation after 1h of illumination (beginning of day period); presented data are representative of three independent experiments; B – Western blot immunodetection of STN8 kinase (AS10 1601).

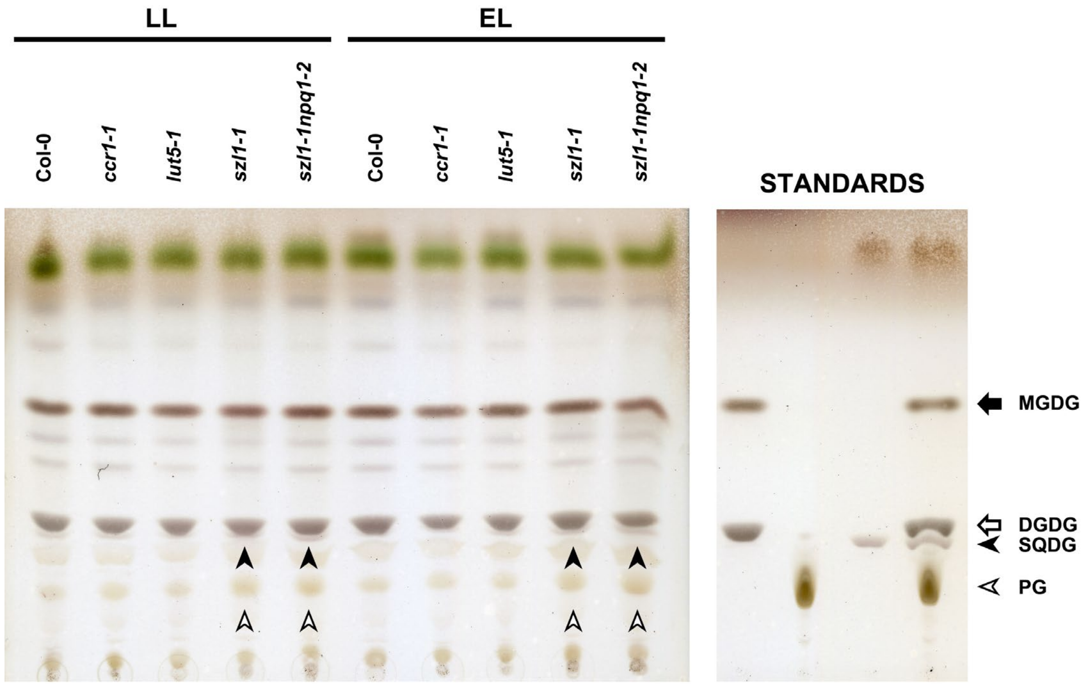

Supplemental Figure S6

Thin layer chromatography (TLC) of total leaf lipid extracts.

Extracts were obtained from Columbia-0 (Col-0) and carotenoid-deficient Arabidopsis plants grown in low light (LL) and adapted to excessive light conditions (EL). TLC plate visualized using H_2SO_4 and the plate with lipid standards developed in the same conditions; MGDG – monogalactosyldiacylglycerol, DGDG – digalactosyldiacylglycerol, SQDG – sulfoquinovosyldiacylglycerol, PG – phosphatidylglycerol; presented data are representative of three independent experiments. Black and white arrowheads on the left hand side indicate increased levels of SQDG an PG, respectively.

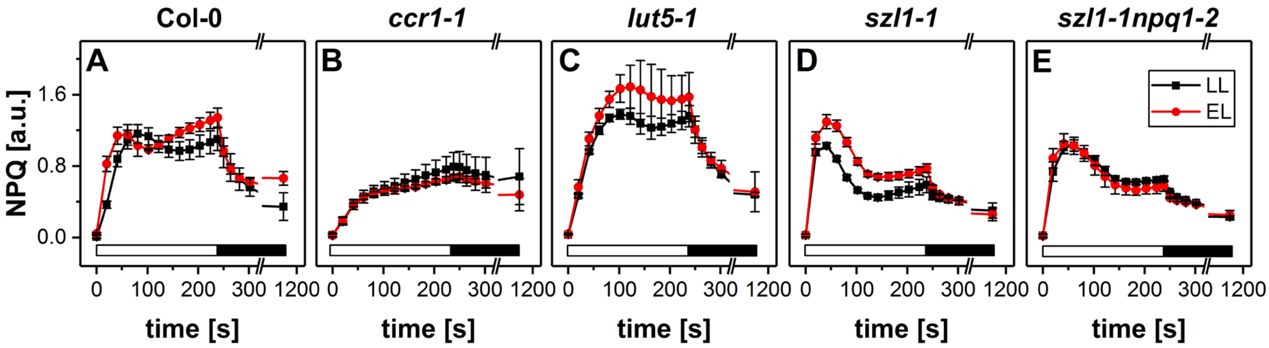

Supplemental Figure S7

Non-photochemical quenching (NPQ) measurements.

Experiments were performed on Columbia-0 (Col-0) and carotenoid-deficient Arabidopsis plants grown in low light (LL) and adapted to excessive light conditions (EL). A-E – changes of NPQ during illumination of dark-adapted plants under high-light conditions and dark recovery phase; details of the measurement protocol are described in Podgórska et al. 2020. Data are mean values ± SD from at least three independent experiments.

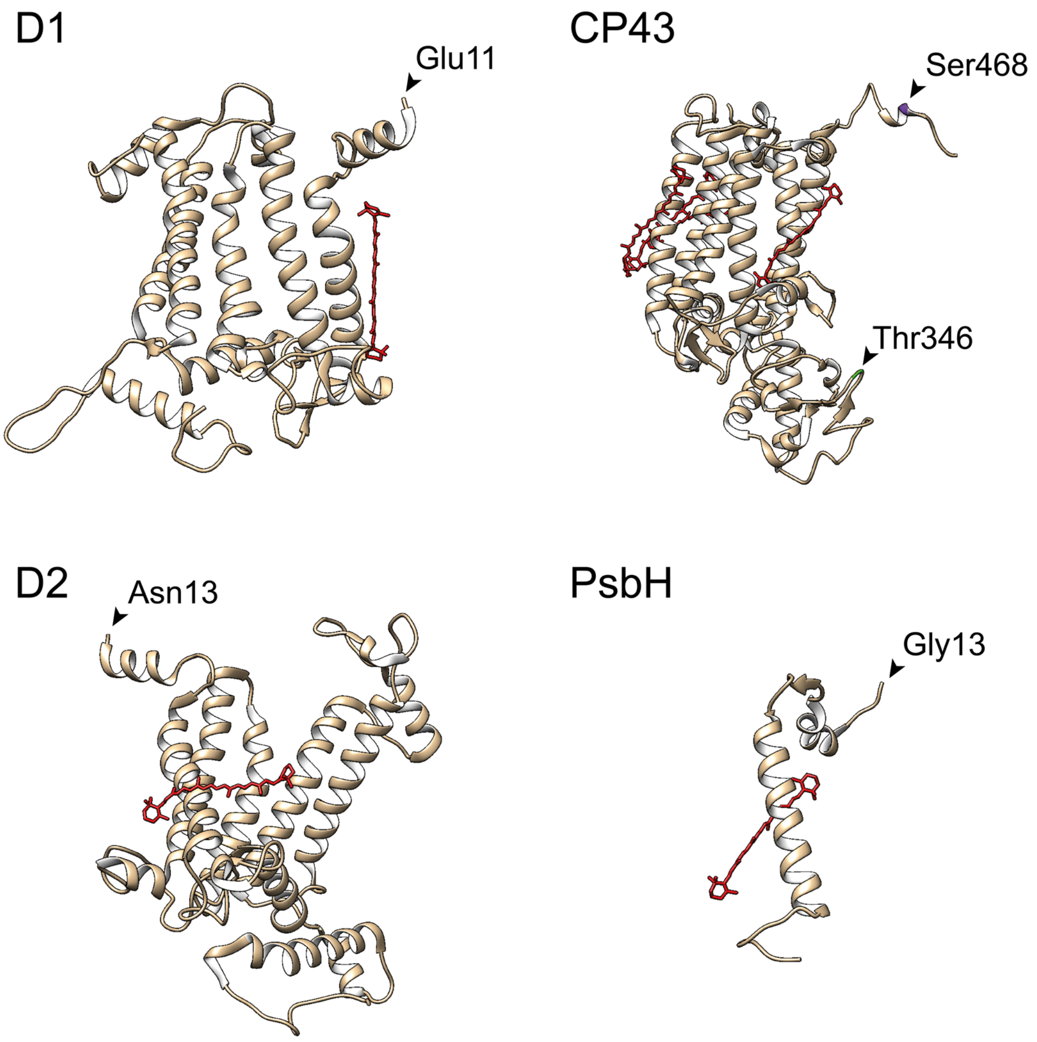

Supplemental Fig. S8

Photosystem II (PSII) protein models showing β-carotene localization and STATE TRANSITION 8 (STN8) kinase phosphorylation sites.

Models of PSII proteins generated on the basis of pea PSII supercomplex crystal (Su et al. 2017) showing directly the particular protein phosphorylation site (Ser468 and Thr346 in CP43 protein) or position of amino acid determined in the model which is the closest one to the missing residue where the confirmed phosphorylation site of a particular protein is located (Thr2 in D1 and D2, Thr3 and Thr5 in PsbH) (Pesarasi *et al.* 2011). Molecular graphics and analyses were performed with UCSF Chimera, developed by the Resource for Biocomputing, Visualization, and Informatics at the University of California, San Francisco (Pettersen et al. 2004), with support from NIH P41-GM103311.

Supplemental Table S1

Carotenoid and antioxidant component ratios.

Experiments were performed on leaf-extracts of Columbia-0 (Col-0) and carotenoid-deficient Arabidopsis plants grown in low light (LL) and adapted to excessive light conditions (EL). Chl – chlorophyll, Lut – lutein, Car – carotene, α-T – α-tocopherol, PQ – plastoquinone; results are present as mean values ± SD from at least three independent experiments, pairs of results marked with different letter differ significantly at *p* = 0.05 (one-way ANOVA with post-hoc Tukey test).

	**LL**					**EL**				
**Compound ratios**	**Col-0**	***ccr1-1***	***lut5-1***	***szl1-1***	***szl1-1 npq1-2***	**Col-0**	***ccr1-1***	***lut5-1***	***szl1-1***	***szl1-1 npq1-2***
					Carotenoid to Chl ratio					
**Carotenoids/ Chl**	0.169 ± 0.003[ac]	0.174 ± 0.001[ab]	0.169 ± 0.002[c]	0.175 ± 0.004[ab]	0.191 ± 0.001[d]	0.173 ± 0.002[ac]	0.179 ± 0.002[b]	0.178 ± 0.005[b]	0.185 ± 0.001[d]	0.191 ± 0.001[d]
					Carotenoid ratios					
**Lut/β-Car**	1.92 ± 0.01[a]	1.00 ± 0.48[a]	3.06 ± 0.09[a]	11.85 ± 1.98[b]	7.51 ± 0.73[c]	2.02 ± 0.09[b]	1.17 ± 0.33[a]	1.98 ± 0.39[a]	9.03 ± 0.61[c]	9.29 ± 0.70[c]
**Lut/Car total**	1.78 ± 0.02[ab]	0.98 ± 0.47[b]	1.33 ± 0.04[ab]	3.98 ± 0.62[c]	4.01 ± 0.14[c]	1.92 ± 0.09[a]	1.15 ± 0.32[b]	1.67 ± 0.33[ab]	3.16 ± 0.27[d]	3.86 ±0.36[cd]
**α-Car/β-Car**	0.08 ± 0.01[adf]	0.02 ± 0.00[a]	1.31 ± 0.27[b]	0.67 ± 0.05[ce]	0.38 ± 0.06[de]	0.05 ± 0.02[adf]	0.02 ± 0.00[ad]	0.19 ± 0.16[adf]	0.58 ± 0.27[ce]	0.39 ±0.06[ef]
**Lut/ββ-xanthophylls**	1.51 ± 0.17[a]	0.62 ± 0.15[a]	2.74 ± 0.21[a]	9.35 ± 3.07[b]	10.50 ± 1.20[b]	1.69 ± 0.09[a]	2.90 ± 0.14[a]	2.29 ± 0.34[a]	9.45 ± 3.09[b]	9.77 + 0.22[b]
					Antioxidant component ratio					
**(α-T+PQ+Car total)/(α-T+PQ+ carotenoids)**	0.31 ± 0.03[a]	0.45 ± 0.05[bc]	0.40 ± 0.07[ac]	0.45 ± 0.01[ab]	0.40 ± 0.02[ac]	0.46 ± 0.13[bc]	0.57 ±0.04[b]	0.46 ± 0.01[bc]	0.41 ±0.03[ac]	0.47 ± 0.05[bc]

Supplemental data – literature cited:

Alagoz Y, Dhami N, Mitchell C, Cazzonelli CI (2020) cis/trans Carotenoid extraction, purification, detection, quantification, and profiling in plant tissues. Methods Mol Biol 2083: 145-163

Pettersen EF, Goddard TD, Huang CC, Couch GS, Greenblatt DM, Meng EC, Ferrin TE (2004) *J* UCSF Chimera--a visualization system for exploratory research and analysis. Comput Chem **13:** 1605-2612

Podgórska A, Mazur R, Ostaszewska-Bugajska M, Kryzheuskaya K, Dziewit K, Borysiuk K, Wdowiak A, Burian M, Rasmusson AG, Szal B (2020) Efficient photosynthetic functioning of arabidopsis thaliana through electron dissipation in chloroplasts and electron export to mitochondria under ammonium nutrition. Front Plant Sci 11: 103

Su X, Ma J, Wei X, Cao P, Zhu D, Chang W, Liu Z, Zhang X, Li M (2017) Structure and assembly mechanism of plant C2S2M2-type PSII-LHCII supercomplex. Science **357:** 815-82
