# Supplementary figures and images for: Too rigid to fold: Carotenoid-dependent decrease in thylakoid fluidity hampers the formation of chloroplast grana
Source: Plant Physiol. 2020 Nov 28;185(1):210–27. doi: 10.1093/plphys/kiaa009 (PMC8133577; doi:10.1093/plphys/kiaa009)

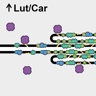

Supplement: kiaa009_Supplementary_Data [file kiaa009_supplementary_data.zip › kiaa009-suppl_data/pp.01286.2020-s02.jpg]
